# A small-scale proteomic approach reveals a survival strategy, including a reduction in alkaloid biosynthesis, in *Hyoscyamus albus* roots subjected to iron deficiency

**DOI:** 10.3389/fpls.2013.00331

**Published:** 2013-08-28

**Authors:** Jebunnahar Khandakar, Izumi Haraguchi, Kenichi Yamaguchi, Yoshie Kitamura

**Affiliations:** ^1^Graduate School of Science and Technology, Nagasaki UniversityNagasaki, Japan; ^2^Graduate School of Fisheries Science and Environmental Studies, Nagasaki UniversityNagasaki, Japan; ^3^Division of Biochemistry, Faculty of Fisheries, Nagasaki UniversityNagasaki, Japan

**Keywords:** *Hyoscyamus albus*, roots, Fe deficiency, small-scale proteomics, hyoscyamine 6β-hydroxylase, tropane alkaloid biosynthesis, malic acid secretion

## Abstract

*Hyoscyamus albus* is a well-known source of the tropane alkaloids, hyoscyamine and scopolamine, which are biosynthesized in the roots. To assess the major biochemical adaptations that occur in the roots of this plant in response to iron deficiency, we used a small-scale proteomic approach in which 100 mg of root tips were treated with and without Fe, respectively, for 5 days. Two-dimensional mini gels showed that 48 spots were differentially accumulated between the two conditions of Fe availability and a further 36 proteins were identified from these spots using MALDI-QIT-TOF mass spectrometry. The proteins that showed elevated levels in the roots lacking Fe were found to be associated variously with carbohydrate metabolism, cell differentiation, secondary metabolism, and oxidative defense. Most of the proteins involved in carbohydrate metabolism were increased in abundance, but mitochondrial NAD-dependent malate dehydrogenase was decreased, possibly resulting in malate secretion. Otherwise, all the proteins showing diminished levels in the roots were identified as either Fe-containing or ATP-requiring. For example, a significant decrease was observed in the levels of hyoscyamine 6β-hydroxylase (H6H), which requires Fe and is involved in the conversion of hyoscyamine to scopolamine. To investigate the effects of Fe deficiency on alkaloid biosynthesis, gene expression studies were undertaken both for H6H and for another Fe-dependent protein, Cyp80F1, which is involved in the final stage of hyoscyamine biosynthesis. In addition, tropane alkaloid contents were determined. Reduced gene expression was observed in the case of both of these proteins and was accompanied by a decrease in the content of both hyoscyamine and scopolamine. Finally, we have discussed energetic and Fe-conservation strategies that might be adopted by the roots of *H. albus* to maintain iron homeostasis under Fe-limiting conditions.

## Introduction

The tropane alkaloids, hyoscyamine and scopolamine, are secondary metabolites produced by some members of the Solanaceae family, such as *Atropa, Datura, Duboisia*, and *Hyoscyamus*. These alkaloids have anticholinergic effects and, therefore, atropine (*dl*-hyoscyamine) and scopolamine are used as mydriatics and analgesics, respectively (Evans, 1996). Tropane alkaloid biosynthesis has been intensively studied, and it is well known that these compounds are biosynthesized in the roots and then transported to the aerial parts of the plant (Manske and Holmes, 1965). Biosynthesis begins with the conversion of putrescine to *N-methylputrescine*, catalyzed by putrescine *N-methyltransferase* (Walton et al., 1994; Biastoff et al., 2009) and ultimately leads to the end-product, scopolamine, which is generated from hyoscyamine by the bi-functional enzyme, hyoscyamine 6β-hydroxylase (H6H) (Hashimoto et al., 1993). The important step to produce hyoscyamine from littorine, *via* a molecular rearrangement catalyzed by a cytochrome P450 enzyme (Cyp80F1) (Li et al., 2006) has recently been investigated at the gene-expression level. Since tropane alkaloids are commercially important plant-derived drugs, manipulation of their biotechnological production using hairy-root cultures or by metabolic engineering has been actively investigated (Zeef et al., 2000; Rahman et al., 2006; Wilhelmson et al., 2006; Zhang et al., 2007). Nevertheless, many important aspects of their biosynthesis, especially in relation to developmental and environmental factors, remain poorly understood.

Iron (Fe) availability is one of the major nutrient constraints for plant growth and development, especially in neutral and alkaline soils, owing to the low solubility of Fe (Lindsay and Schwab, 1982). Insufficient levels of Fe induce a range of morphological and metabolic changes required to withstand the resultant stress and to maintain Fe homeostasis (Thimm et al., 2001; Zaharieva et al., 2004). Higher plants take up Fe through their roots, so that Fe deficiency initially and most directly affects the roots; and therefore survival under Fe deficiency depends upon the root system, although aerial parts also suffer from serious damage (Rodríguez-Celma et al., 2013a). Using a hairy-root culture system of *H. albus*, we have found that cultured roots are able to grow under Fe deficiency, although the roots show morphological changes, such as shorter, swollen root tips, that have been observed also in the roots of other plants (Rodríguez-Celma et al., 2011a). Interestingly, *H. albus* roots secrete flavin (riboflavin) into the rhizosphere under these conditions (Higa et al., 2008, 2010), in the same way as other, taxonomically unrelated, dicotyledonous plants, including *Beta vulgaris* (Susin et al., 1994), *Medicago truncatula* (Rodríguez-Celma et al., 2011a,b), *Cucumis sativus* (Shinmachi et al., 1997) and *Helianthus annuus* (Raju and Marschner, 1973).

In order to address the range of metabolic and respiratory adaptations of *H. albus* hairy roots to Fe deficiency, we have initially investigated the characteristics of mitochondrial respiration in these roots, and especially their electron transport chains (ETC) (Higa et al., 2010). The plant mtETC consists of complex I to complex IV, which are components found in all organisms (Dudkina et al., 2006), in addition to a plant-specific alternative oxidase (AOX) and NAD(P)H dehydrogenases (ADX). During electron transport from complex I to complex IV, proton gradients are generated, resulting in the synthesis of ATP, the universal energy currency, through the action of ATP synthase (complex V). Our feeding experiments with respiratory-component-specific inhibitors have indicated that the mtETC changes in response to Fe deficiency (Higa et al., 2010): under these conditions, electrons mainly flow through the alternative dehydrogenase (ADX) to complexes III and IV, whereas both complexes I and II and the AOX are less active. It is noteworthy that complexes I and II contain a large number of Fe ions, whilst AOX does not contribute to the generation of a proton gradient (Ohnishi, 1998; Taiz and Zeiger, 2002; Vigani et al., 2009). On this basis, we have proposed that riboflavin secretion occurs as a result of the underuse of flavoprotein complexes I and/or II (Higa et al., 2010), although both increased *de novo* riboflavin synthesis and hydrolysis of FMN could be involved in riboflavin secretion (Higa et al., 2012). On the other hand, it has been proposed that flavins accumulated in the roots may act as electron donors or as cofactors for Fe (III) reductase (López-Millán et al., 2000; Rodríguez-Celma et al., 2011a,b), because the Fe reductase contains FAD as a cofactor (Schagerlöf et al., 2006). Very recently, Rodríguez-Celma et al. (2013b) proposed a hypothesis that flavins function as Fe-binding compounds in the utilization from usually unavailable Fe pools. In spite of several possible hypotheses including those mentioned above, the actual cause and function of secreted/accumulated flavins under Fe deficiency remain uncertain.

As outlined above, our results have indicated that the mtETC machinery undergoes a shift to a less Fe-dependent mode under Fe-deficient conditions. We contend that this adaptation is likely to be a more extensive phenomenon involving wide-ranging adaptations at the cellular and tissue levels. To explore this idea, we decided to undertake a global protein expression survey using a proteomic approach. Proteomics has become a powerful tool to study many aspects of plant biology, including Fe-deficiency stress (López-Millán et al., 2013). Both in model plants such as *Arabidopsis* (Lan et al., 2011) and *Medicago* (Rodríguez-Celma et al., 2011a), and in crop plants such as *B. vulgaris* (Rellán-Alvarez et al., 2010), *C. sativus* (Donnini et al., 2010), *Prunus* hybrid (Rodríguez-Celma et al., 2013a), and *Lycopersicum esculentum* (Brumbarova et al., 2008; Li et al., 2008), adaptations to Fe deficiency have been examined by a proteomic approach. However, there have been very few investigations reported with medicinal plants, many of which are technically less amenable to study (Aghaei and Komatsu, 2013). Furthermore, most studies have used medium- or large-scale extractions and required large amounts of sample, which are often unavailable for less amenable plant species, or when very detailed, organ- or tissue-specific analyses need to be made. We faced precisely these difficulties in undertaking the proteomic analysis of cultured root tips of *H. albus*; the amount of tissue available was very small and no proteomic analyses had been reported for any *Hyoscyamus* spp. Here we report the application of a small-scale proteomics for comparison of protein profiles from *H. albus* root tips subjected to two different Fe statuses.

## Materials and methods

### Root culture and sample collection

Hairy roots of *Hyoscyamus albus* L. (Solanaceae) used in these experiments had been established previously (Higa et al., 2008). Roots were maintained on MS basal liquid medium (Murashige and Skoog, 1962) containing 3% sucrose. A primary root tip with a few lateral roots (*ca*. 2 cm in length) isolated from ca. 2-week-old root cultures was pre-propagated in the normal liquid B5 medium (Gamborg et al., 1968), containing 1% sucrose, for 2 weeks. After pre-propagation, root cultures were separated into sub-sets by exchanging the medium for fresh B5 medium containing 1% sucrose, either with Fe or without Fe; culture was then continued for 5 days. Fe deficiency stress was imposed by the elimination of Fe(III)-EDTA (100 μM in the final concentration) from B5 basal medium, prior to autoclaving at 121°C for 15 min. All cultures were performed in 100 mL conical flasks containing 25 mL of liquid medium and incubated at 25°C with agitation at 80 rpm on a rotary shaker (SHK-420N Iwaki, Tokyo, Japan) under the sterile conditions until harvest. For protein and transcript analyses, root tips were harvested at day 5, frozen in liquid nitrogen and stored at −80°C until further use. In the case of alkaloid analysis, the roots cultured ±Fe for 5 days were harvested by vacuum filtration. The root tips were then excised and the tips and the remaining parts of the roots were separated and each dried to constant weight at 50°C, before being ground to a powder using a mortar and pestle. The culture medium was also collected for 5 days and analyzed for organic acids. All analyses were carried out by HPLC using three biological replicates (more details were in Alkaloid Extraction and Analysis and Organic acid Analysis).

### Protein extraction

Protein was extracted from root tips using bead-beating, followed by acid guanidinium-phenol-chloroform treatment (the optimization of this protocol to *H. albus* roots and its effectiveness will be reported elsewhere). A 100 mg (fresh weight) of frozen root tips, together with 20% w/w dithiothreitol (DTT, Bio-Rad, Hercules, CA, USA) and 10% w/w polyvinylpolypyrrolidone (PVPP, Sigma-Aldrich, St. Louis, MO, USA), and one stainless-steel bead crusher (SK-100-D10, Tokken, Inc., Chiba, Japan) were placed in the 2-mL stainless-steel tube (Tokken, Inc., Chiba, Japan). The tubes were then mounted in the Master Rack aluminum block (BMS, Tokyo, Japan) under liquid nitrogen and agitated for 1 min in the ShakeMaster Auto ver 1.5 (BMS, Tokyo, Japan). To the tube, 1 mL of TRIzol reagent (Invitrogen, Boston, MA, USA) was added and protein extraction was performed in accordance with manufacturer's instructions. The resulting pellet was then incubated with 1 mL ethanol for 20 min at room temperature, the solution was centrifuged (7500 × g, 5 min, at 4°C), and the pellets were dried for 5–10 min on the bench with the centrifuge tube lids open. To each pellet, 100 μL of resuspension solution (8 M urea, 50 mM DTT, 2% w/v CHAPS, 0.2% v/v carrier ampholyte, 0.001% w/v bromophenol blue) was added; then the mixture was homogenized with a plastic pestle, and sonicated for 1 min (10 s × 6; output level 4) in the ice-cold cup of a horn-type sonicator (Astrason Ultrasonic Processor XL2020, Misonix, NY, USA) and finally incubated for 1 h at room temperature. Protein concentration in the solution was determined by a modified Bradford assay (Quick Start® Protein Assay Kit, Bio-Rad), using bovine serum albumin (BSA) as a standard.

### 2-D gel electrophoresis

The first dimensional iso-electrofocusing (IEF) separation was carried out using 7-cm ReadyStrip® IPG Strips (linear pH gradient, pH 5–8, Bio-Rad) using a Protean® IEF Cell (Bio-Rad,). The IPG strips were passively rehydrated for 12 h at 20°C in 125 μL of resuspension solution (8 M urea, 50 mM DTT, 2% w/v CHAPS, 0.2% v/v carrier ampholyte, 0.001% w/v bromophenol blue), containing protein sample (20 μg/strip). IEF was carried out at 20°C, for a total of 20,000 Vh (15 min with a 0–250 V linear gradient; 2 h with a 250–4000 V linear gradient; and finally 4000 V, held constant until 20,000 Vh had been reached). After IEF, proteins in the strips were reduced and alkylated by gentle stirring for 10 min in equilibration buffer (6 M urea, 0.375 M Tris-HCl, pH 8.8, 2% w/v SDS, 20% v/v glycerol), supplemented with 2% w/v DTT, and for an additional 10 min in the same equilibration buffer supplemented with 2.5% w/v iodoacetamide. The second dimensional electrophoresis was performed with the Mini-PROTEAN Tetra® electrophoresis cell (Bio-Rad, Hercules, CA, USA). The equilibrated IPG strips were placed on top of Mini-Protean TGX® precast gels (AnykD IPG/Prep®, Bio-Rad), sealed with ReadyPrep® overlay agarose (Bio-Rad) and electrophoresed at 200 V in 25 mM Tris base, 192 mM glycine and 0.1% w/v SDS, for *ca*. 30 min at room temperature. Gels were stained with Flamingo® fluorescent stain (Bio-Rad) and the gel images for figure presentation were captured using a GELSCAN® laser scanner (iMeasure, Nagano, Japan).

### Image analysis, spot detection and statistical analysis

For 2-DE pattern analyses, four biological replicates for each condition and four technical replicates for each protein sample were compared between Fe-replete and Fe-deficient conditions. Differences in spot abundance were statistically evaluated by ANOVA (*p* < 0.05) after normalization, using the Prodigy SameSpots® software package (Non-linear Dynamics, Newcasle, UK). A criterion of an abundance ratio (≥1.5-fold) was used to define significant differences.

### Protein in-gel digestion and MALDI-QIT-TOF/MS analysis

Protein spots were manually excised from 2D-gels using a spot image analyzer (FluoroPhoreStar 3000®, Anatech, Tokyo, Japan) equipped with a gel picker (1.8-mm diameter). In-gel tryptic digestion and peptide extraction were carried out as previously described by Yamaguchi (2011). The dried samples were dissolved in 2 μL of DHBA solution (5 mg mL^−1^ of 2, 5-dihydroxybenzoic acid, in 33% v/v acetonitrile and 0.1% v/v trifluoroacetic acid), and 1 μL samples of the solutions were spotted onto a stainless 384-well MALDI target plate (Shimadzu GLC, Tokyo, Japan). For peptides obtained from faint spots, the dried sample was dissolved in 2 μL of 1 10^−1^ diluted DHBA solution, and 1 μL of the solution was spotted onto a μFocus MALDI target plate (Hudson Surface Technology, NJ, USA). MS and MS/MS spectra were obtained using a MALDI-QIT-TOF mass spectrometer (AXIMA Resonance, Shimadzu, Kyoto, Japan) in the positive mode. All the spectra were externally calibrated using human angiotensin II (m/z: 1046.54) and human ACTH fragment 18–39 (m/z: 2465.20) in a ProteoMass® Peptide and Protein MALDI-MS Calibration Kit (Sigma-Aldrich, St. Louis, MO, USA). MS/MS ion searches were performed using MASCOT® version 2.3 (Matrix Science, London, UK) against SwissProt 2012_06 (536,489 sequences; 190,389,898 residues), EST_Solanaceae 2012_04 (2,984,694 sequences; 560,101,884 residues) and NCBInr 20130614 (26236801 sequences; 9088244489 residues) in our own MASCOT server. Search parameters used were: enzyme, trypsin; fixed modifications, carbamidomethyl (Cys); variable modifications, oxidation (H, W, and M); mass values, monoisotopic; peptide mass tolerance, ± 0.5 Da; fragment mass tolerance, ± 0.5 Da, max missed cleavage, 1. Positive identification was assigned with Mascot scores above the threshold level (*p* < 0.05), at least two peptide (protein score > 44) or single peptide (peptide score > 47), 2% sequence coverage, and similar theoretical and observed mass and *pI* values. In the case of single peptide identification, annotated MS/MS spectra of Mascot search results (peptide view) were shown in Supplemental data 2.

### Determination of *pI*, MW and subcellular localization

Theoretical isoelectric points (*pI*) and sequence mass of the precursor protein were calculated using ProtParam (http://www.expasy.ch/tools/protparam.html). Observed *pI* was calculated from the horizontal migration of the spot. Observed mass was estimated from the vertical migration of the spot, according to Weber and Osborn (1969). Subcellular location of proteins was predicted by WoLF-PSORT (http://wolfpsort.org/), if it has not been reported.

### RNA extraction and semi-quantitative RT-PCR

Total RNA was isolated from frozen roots of *H. albus* (100 mg) using an RNeasy® Plant Mini Kit (Qiagen, Tokyo, Japan) and following the manufacturer's instructions. The concentrations of RNAs were determined using a NanoDrop ND-1000 spectrometer (NanoDrop Technologies, DE, USA). For the analysis of the expression of genes involved in tropane alkaloid biosynthesis, we focused on the genes encoding the enzymes H6H and Cyp80F1. Primers for *H6H* were designed by the alignment of conserved cDNA sequences of *H. niger* and other tropane alkaloid-producing plants, previously deposited in the database (GenBank accessions, M62719 and EU530633). In the case of Cyp80F1, primer pairs were used, according to the previous report (Li et al., 2006). The expression of *riboflavin synthase* (*RibD*) was also determined as a representative gene involved in riboflavin biosynthesis, according to our previous report (GenBank accession, AB712370) (Higa et al., 2012). Primers used were as follows: *H6H*, forward (5′-GGTCTCTTTCAGGTGATCAA-3′) and reverse (5′-CTTCACAGATGTAGTCCAGCA-3′); *Cyp80F1*, forward (5′-CACAGTTGAATGGACATTGGTGGAGC-3′) and reverse (5′-GAACAGTAATGGCGCCGGAGGATGC-3′); *RibD*, forward (5′-GTTGTCGGAATTTAGTGTCGG-3′) and reverse (5′-TCCCAGTCTTGACCTTCACC-3′). The universal primer pairs for 18S ribosomal RNA were used as a control for semi-quantification, according to the manufacture's recommendations (Applied Biosystems, CA, USA). RT-PCR was performed using an mRNA-selective PCR kit (Takara Bio, Shiga, Japan) with the primers mentioned above. A sample aliquot containing 1.0 μg RNA for both *H6H* and *Cyp80F1* or 0.5 μg RNA for *RibD* was subjected to reverse transcription (25 min for *H6H* and *Cyp80F1*; 30 min for *RibD*, at 45°C). The PCR conditions were, as follows: 1 min at 85°C, 1 min at 45°C, 1 min at 72°C, 23 cycles for *H6H* and *Cyp80F1*; 1 min at 85°C, 1 min at 47°C, 1 min at 72°C, 21 cycles for *RibD*. RT-PCR products were loaded onto 3% (w/v) agarose gels and stained with ethidium bromide. A 100-bp DNA ladder (Takara) was used as a molecular marker. Pictures were taken with a gray-scale digital camera (CFW-1310M, Scion Corp., MD, USA) and band intensities were measured using Image J software (NIH, MD, USA).

### Alkaloid extraction and analysis

Alkaloids were extracted from dried root tissues (*ca*. 50 mg) according to the reported method (Sauerwein and Shimomura, 1991). Alkaloid analysis was performed by HPLC as described in a previous report (Hank et al., 2004), with some modifications. We added 5 mM homatropine (60 μL) to extracts at the initial stage, as an internal standard, and the MeOH extracts (20 μL) were applied to an HPLC system (Shimadzu LC-10, Kyoto, Japan) fitted with a Wakosil-II 5C8 RS column (4.6 × 150 mm, Wako Corporation, Osaka, Japan). The eluent conditions were as follows: flow rate, 1 mL min^−1^; column temp, 40°C; solvent A; 30 mM phosphate buffer (pH 6.2), containing 0.1% triethylamine, and MeOH 75/10 (v/v); solvent B, CH_3_CN; isocratic elution, with A: B = 80: 20. Alkaloids were detected at 210 nm with a UV detector (Shimadzu SPD-10AVP) and a photodiode array (Shimadzu SPD-M20A). Quantification was based on standard curves using the reference standard and the internal standard.

### Organic acid analysis

Organic acids in the culture medium, including malic acid and citric acid, were measured by HPLC (Intelligent HPLC system, Jasco, Tokyo, Japan), according to the reported method (Nisperos-Carriedo et al., 1992). Before applying, insoluble particles in the culture medium were removed with 0.2 μm membrane filters (Millex-LG, Merck Millipore, MA, USA) and then 10–20 μL eluent was applied to an Inertsil ODS-3 column (4.6 × 150 mm, GL sciences Inc, Japan). The HPLC conditions were as follows: flow rate, 0.5 mL min^−1^; column temp, 25°C; detection, 220 nm; solvent A, MeOH; solvent B, 2% NaH_2_PO_4_ (pH 2.3, adjusted with H_3_PO_4_); isocratic elution, with A: B = 2: 98. Quantification was based on standard curves established with standard compounds.

## Results and discussion

### Small-scale proteomic approach

Since the effects of Fe deficiency appear prominently in root tips both in our system and in other plant systems (Rellán-Alvarez et al., 2010), we decided to collect root tips for proteomic analysis. In order to eliminate bacterial contamination or other environmental factors that can affect plant metabolism and to guarantee reproducible data, we used sterile root cultures as our source material. Our previous study had shown that both flavin mononucleotide (FMN) hydrolase activity and respiration activity in root tips of *H. albus* were significantly higher at day 5 after transfer to Fe-deficient medium (Higa et al., 2012). We tried to harvest root tips at day 5, cultured in both Fe-deficient and -replete media, for proteomic analysis.

By this method, the total protein amounts soluble in CHAPS buffer that were obtained from Fe-deficient and Fe-replete samples, respectively, were 5.57 ± 0.56 and 4.92 ± 0.60 mg g^−1^ fr. wt (from four biologically independent experiments). Fe-deficient root tips contained slightly higher amounts of protein (by *ca*. 13%) than Fe-replete ones (Table 1).

Table 1**Summarized results of protein yields and profiles from *H. albus* root tips**.

**Table:** 

**Protein yield and spot number**	**+Fe**		**−Fe**
Root tips (mg FW)	100		100
Protein yields (mg protein g^−1^FW)	4.92 ± 0.60		5.57 ± 0.56
No. of spots detected	218 ± 4		219 ± 4

**Table:** 

**Comparative protein profile**	**−Fe/+Fe**	
No. of spots changed^*^	48	
(Increased in abundance)		(30)
(Decreased in abundance)		(18)
No. of spots identified	36	
(Increased in abundance)		(22)
(Decreased in abundance)		(14)

Twenty μg of proteins extracted from 100 mg of H. albus root tips cultured under Fe-replete and Fe-deficient conditions for 5 days were separated on small gels (7 × 7 cm).

* To analyze differential protein accumulation between the two conditions of Fe availability, a threshold value (ANOVA at p < 0.05) was set at ≥1.5-fold change after normalization using Prodigy SameSpots®.

### Changes in the protein profile under iron deficiency

**Figure 1:**
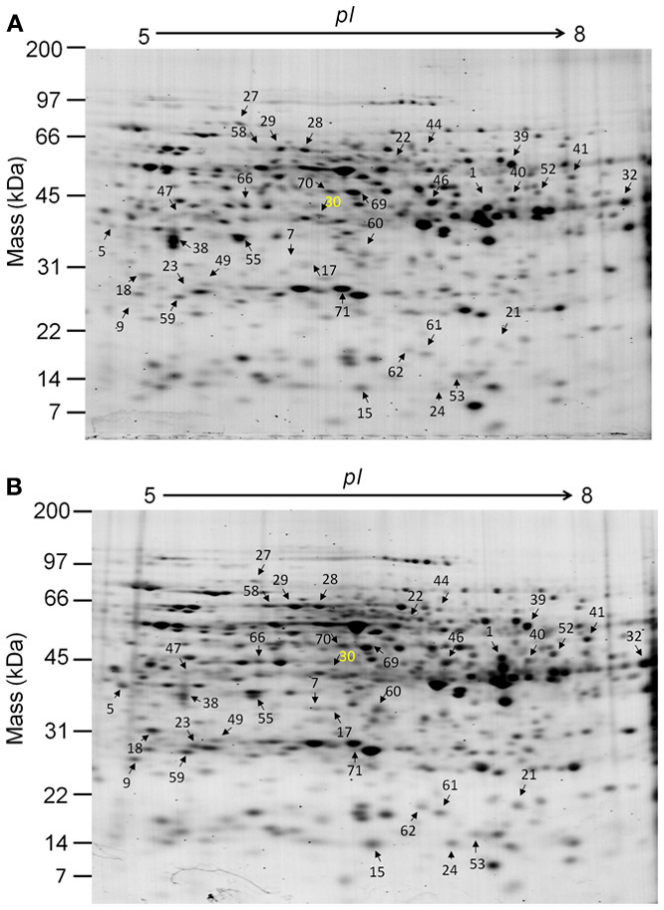
**2-D Gel electrophoretic separations of *Hyoscyamus albus* root-tip proteins extracted from cultures exposed to Fe-replete (A) and Fe-deficient (B) conditions.** Twenty μg of proteins extracted from 100 mg of *H. albus* root tips cultured under Fe-replete **(A)** and Fe-deficient **(B)** conditions for 5 days were separated on 7 cm IPG strips (pH5-8 linear gradient) using isoelectric focusing (IEF) in the first dimension, followed by AnyKd® gels in the second dimension. Gels were stained with Flamingo fluorescent stain. The prodigy rank number was used as a spot number; the number shown in yellow color is identified as hyoscyamine 6β-hydroxylase (H6H), the others are shown in black color. Molecular mass (kDa) and *pI* are indicated on the left-hand and upper axes, respectively.

Using our small-scale proteomic approach, 219 ± 4 spots and 218 ± 4 spots (from four biological samples, analyzed in four times) were resolved from protein extracts obtained from Fe-deficient and Fe-replete root tips, respectively (Figure 1; Table 1). To analyze differential protein accumulation between the two conditions of Fe availability, we first set ≥1.5-fold change as a threshold value (ANOVA at *p* < 0.05) after normalization using Prodigy SameSpots®. Using these parameters, a total of 48 spots were detected; of these, 30 were increased in abundance and 18 were decreased under Fe deficiency (Table 1).

After in-gel digestion of each protein spot, followed by peptide extraction, the solutions were applied to matrix-assisted laser desorption/ionization–quadrupole ion trap–time of flight (MALDI-QIT-TOF) mass spectrometry for analysis of MS/MS. In this way, a total of 36 proteins were identified (Tables 2, 3; Supplemental data 1 and 2); of these, 22 were increased in abundance, while the others were decreased under Fe deficiency (Figure 1; Tables 1–3). The results of this small-scale proteomic analysis revealed that most of the identified proteins have already been reported in the previous proteomic studies, but 10 proteins seemed not to be matched to the previously identified proteins from other dicotyledonous plants under Fe deficiency (Li et al., 2008; Donnini et al., 2010; Rellán-Alvarez et al., 2010; Wang et al., 2010; Lan et al., 2011; Rodríguez-Celma et al., 2011a, 2013a). These proteins included chaperonin 21 (spot 9), peroxidase 27 putative (spot 1), peroxiredoxin (spot 62), galactose oxidase/kelch repeat-containing protein (Glx, spot 5), elongation factor Tu (spot 46), aspartic protease (spot 60), soluble inorganic pyrophosphatase (spot 49), sinapyl alcohol dehydrogenase (spot 66), hyoscyamine 6β-hydroxylase (H6H, spot 30), and acetoacetyl-CoA thiolase (spot 52) (Figure 1; Tables 2, 3). Some of these proteins, like H6H, could be highly dependent on plant species and materials. However, the comparison of proteomes from various plant species is not simple, because protein identification is restricted to the availability of public database and its reliability depends on the stringency of the criteria selected for positive identification at least (López-Millán et al., 2013).

**Table 2:** **MS/MS-based cross-species identification and characterization of the protein spots that showed significant volume increase under iron-deficient conditions**.

**Spot No.**	**Protein name**	**Species**	**Accession no (NCBI)**	**Theoretical mass (kDa)/pI**	**Observed mass (kDa)/pI**	**PS/NMP^†^**	**SC (%)^‡^**	**Fold (−Fe/+Fe)**	**Subcellular localization**
**CARBOHYDRATE METABOLISM**									
58	UDP-Glucose pyrophosphorylase	*Solanum tuberosum*	P19595	51.87/5.70	77.8/6.2	52/1^a^	7^b^	1.7	Cytoplasm
28	Phosphoglycerate mutase	*Solanum tuberosum*	AAD24857.1	61.26/5.42	77.8/6.3	64/3	6	1.8	Cytoplasm
29	Phosphoglycerate mutase	*Solanum tuberosum*	AAD24857.1	61.26/5.42	76.0/6.3	48/2	3	1.7	Cytoplasm
22	Enolase	*Solanum lycopersicum*	NP_001234080.1	47.79/5.68	47.6/6.7	75/2	8	2.0	Cytoplasm
32	Fructose bisphosphate aldolase like protein	*Solanum tuberosum*	ABB29926.1	38.53/8.32	45.4/7.9	53/2	11	1.7	Cytoplasm
41	Fumarase	*Solanum tuberosum*	CAA62817.1	53.38/6.51	60.7/7.7	56/1^a^	2	1.6	Mitochondria
**DEFENSE RESPONSE**									
7	Pyridoxine biosynthesis protein isoform A	*Lotus japonicus*	AAZ67141.1	33.09/5.92	41.2/6.3	57/2	9	2.9	Cytoplasm
9	Chaperonin 21 precursor	*Solanum lycopersicum*	NP_001234423.1	26.56/6.85	29.4/5.3	63/2	9	2.8	Plastid
1	Peroxidase 27 precursor putative	*Ricinus communis*	XP_002527239.1	35.67/8.89	46.5/7.1	52/1^a^	5	9.4	Secreted
62	Peroxiredoxin	*Ipomoea batatas*	AAP42502.1	20.76/8.80	23.1/6.8	94/3	21	1.5	Mitochondria
61	Glutathione peroxidase	*Solanum lycopersicum*	NP_001234567.1	18.84/6.58	20.9/6.8	89/3	21	1.5	Secreted
15	Superoxide dismutase [Cu-Zn]	*Nicotiana plumbaginifolia*	P27082.2	15.23/5.47	16.5/6.5	101/2	20	2.1	Cytoplasm
**STRUCTURE / DEVELOPMENT**									
59	Actin	*Bos taurus*	P62739	42.01/5.24	30.8/5.7	59/2	9^b^	1.5	Cytoplasm
21	Ubiquitin-conjugating enzyme E2 13 variant	*Solanum tuberosum*	ABA40444.1	16.56/6.20	17.3/6.8	79/3	29	2.1	Cytoplasm
53	Actin depolymerizing factor 3	*Gossypium hirsutum*	ABD66505.1	16.11/6.74	17.35/6.8	74/2	14	1.6	Cytoplasm
5	Galactose oxidase/ kelch repeat-containing protein	*Medicago truncatula*	XP_003596164.1	35.49/5.43	35.6/5.3	50/2	8	3.3	Cytoplasm
**AMINO ACID/PROTEIN METABOLISM**									
17	Cysteine protease 14	*Trifolium repens*	AAP32193.1	39.66/5.63	37.4/6.3	49/1^a^	5	2.0	Vacuole
60	Aspartic Protease	*Nicotiana tabacum*	ABG37021.1	54.78/5.53	41.2/6.6	67/2	7	1.5	Secreted
**SECONDARY METABOLISM/OTHERS**									
18	Caffeoyl-CoA-*O*- methyltransferase 6	*Nicotiana tabacum*	Q42945.1	27.79/5.30	32.4/5.5	59/1^a^	4	1.6	Cytoplasm
23	Caffeoyl-CoA-*O*-methyltransferase 1	*Nicotiana tabacum*	O24144	26.96/5.41	32.4/5.6	78/2	8^b^	2.0	Cytoplasm
49	Soluble inorganic pyrophsphatase	*Solanum tuberosum*	Q43187.1	24.26/5.59	34.0/5.7	58/1^a^	8^b^	1.6	Cytoplasm
24	6,7-Dimethyl-8-ribityllumazine synthase	*Nicotiana tabacum*	AAQ04061.1	24.42/8.18 (15.1/5.53)^*^	16.7/6.6	55/1^a^	17^b^	2.0	Plastid

† Protein score (PS) and number of matched peptide (NMP) were obtained from Mascot search.

‡ Percentage of sequence coverage (SC) of identified peptides related to the corresponding sequence in database.

a Annotated MS/MS spectra are provided in Supplemental data.

b Based on SwissProt full sequence identified by Mascot search.

* Due to the presence of significantly long (86 aa) transit peptide in the precursor sequence, theoretical mass/pI of the predicted mature protein is also indicated in parenthesis.

**Table 3:** **MS/MS-based cross-species identification and characterization of the protein spots that showed significant volume decreased under iron-deficient conditions**.

**Spot No.**	**Protein name**	**Species**	**Accession no (NCBI)**	**Theoretical mass (kDa)/pI**	**Observed mass (kDa)/pI**	**PS/NMP^†^**	**SC (%)^‡^**	**Fold (+Fe/−Fe)**	**Subcellular localization**
**CARBOHYDRATE METABOLISM**									
40	Fructose bisphosphate aldolase like protein	*Solanum tuberosum*	ABC01905.1	38.62/7.51	47.6/7.5	66/2	9	1.7	Cytoplasm
55	NAD-dependent malate dehydrogenase	*Nicotiana tabacum*	CAB45387.1	43.31/8.03	41.2/6.0	70/2	7	1.6	Mitochondria
**DEFENSE RESPONSE**									
71	Ascorbate peroxidase	*Nicotiana tabacum*	BAA12918.1	27.45/5.43	32.4/6.5	203/6	33	1.5	Cytoplasm
38	Predicted cationic peroxidase	*Vitis vinifera*	XP_002268412.1	34.25/9.36	43.3/5.7	48/1^a^	4	1.7	Vacuole
**STRUCTURE/DEVELOPMENT**									
47	Annexin p34	*Solanum lycopersicum*	NP_001234104.1	35.80/5.37	35.7/6.0	48/2	9	1.6	Cytoplasm
**AMINO ACID/PROTEIN METABOLISM**									
46	Elongation factor Tu	*Arabidopsis thaliana*	Q9ZT91	49.41/6.25	57.8/6.8	217/5	17^b^	1.6	Mitochondria
70	*S*-adenosylmetionine synthase	*Brassica rapa*	Q5DNB1.1	43.16/5.8	48.2/6.3	50/2	7^b^	1.5	Cytoplasm
69	*S*-adenosylmetionine synthase 2	*Solanum tuberosum*	Q38JH8	42.70/5.6	47.9/6.4	52/2	8^b^	1.5	Cytoplasm
44	Nitrite reductase	*Nicotiana tabacum*	BAD15364.1	65.46/6.67	77.3/7.1	57/2	5	1.6	Mitochondria
**SECONDARY METABOLISM/OTHERS**									
66	Sinapyl alcohol dehydrogenase	*Populus trichocarpa*	XP_002322822.1	38.94/6.23	55.1/6.1	68/2	5	1.5	Cytoplasm
30	Hyoscyamine 6β-hydroxylase	*Atropa belladonna*	AEM91979.1	39.22/5.44	37.4/6.4	64/4	16	1.8	Cytoplasm
52	Acetoacetyl-CoA thiolase	*Nicotiana tabacum*	AAU95618.1	41.27/6.47	55.1/7.3	59/2	7	1.7	Cytoplasm
**ETC/ATP SYNTHESIS**									
27	NADH dehydrogenase Fe-S protein 1	*Solanum tuberosum*	Q43644	79.97/5.87	89.3/6.1	45/2	4^b^	1.6	Mitochondria
39	ATP Synthase subunit alpha	*Celtis yunnanensis*	ADL63175.1	55.05/6.23	66.8/7.3	61/2	8	1.7	Mitochondria

† Protein score (PS) and number of matched peptide (NMP) were obtained from Mascot search.

‡ Percentage of sequence coverage (SC) of identified peptides related to the corresponding sequence in database.

a Annotated MS/MS spectra are provided in Supplemental data.

b Based on SwissProt full sequence identified by Mascot search.

The efficient identification of a diverse range of proteins using only small amounts of protein sample (20 μg per gel) must be a reflection of our particular extraction methodology, coupled with the highly-sensitive MALDI-QIT-TOF MS and MS/MS analysis (detection limit, *ca*. 500 amol).

**Figure 2:**
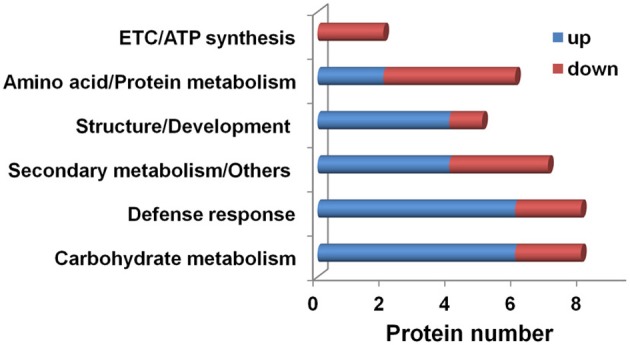
**Classification of differentially accumulated proteins from the root tips of *H. albus* roots cultured under Fe-deficient and Fe-replete conditions.** A total of 36 identified proteins were found to be differentially accumulated and were classified into six functional categories, viz: carbohydrate metabolism; defense response; amino acid/protein metabolism; structure/development; electron transport chains (ETC)/ATP synthesis; and secondary metabolism/others. Blue and red bars indicate up and down proteins in abundance, respectively.

Based on function, 36 differentially accumulated proteins in *H. albus* root tips were roughly classified into six groups: carbohydrate metabolism (8 proteins, 22%), defense response (8 proteins, 22%), structure/development (5 proteins, 14%), amino acid/protein metabolism (6 proteins, 17%), ETC/ATP synthesis (2 proteins, 6%) and secondary metabolism/others (7 proteins, 19%), although there is some overlap between categories. They were further divided into two groups of proteins that were either increased or decreased in abundance, respectively, under Fe deficiency (Figure 2; Tables 1, 2). In the categories of carbohydrate metabolism, defense response and structure/development, 75–80% of proteins identified were increased in abundance. On the other hand, the number of decreased proteins was higher than that of increased proteins in amino acid/protein metabolism. In the case of ETC/ATP synthesis, only decreased proteins were detected (Figure 2).

Our principal findings are now discussed in detail below.

#### Carbohydrate metabolism and malate secretion under Fe deficiency

Our proteomic results showed that the accumulation of a subset of enzymes involved in glycolysis and TCA cycle was increased, including fructose bisphosphate aldolase (spot 32), phosphoglycerate mutase (spots 28, and 29), enolase (spot 22), UDP-glucose pyrophosphorylase (spot 58) and fumarase (spot 41) (Table 2). Indeed, under Fe deficiency, both Fe(III)-chelate reductase (FC-R) and H^+^-ATPase activities are greatly enhanced (Curie and Briat, 2003), leading to a strong demand for energy in the form of NADPH and ATP. In plant cells, the typical energy production process is that hexose or sucrose is metabolized to pyruvate by glycolysis, and then pyruvate is either subjected to fermentation, or fully oxidized via the tricarboxylic acid (TCA) cycle. *H. albus* roots can continue to grow under Fe deficiency (Higa et al., 2008, 2010), so obviously energy generation can be maintained. Enhancement of glycolysis is likely to be the most conspicuous initial indicator of high energy production, as recently reported (Donnini et al., 2010; Rellán-Alvarez et al., 2010; Rodríguez-Celma et al., 2011a).

**Figure 3:**
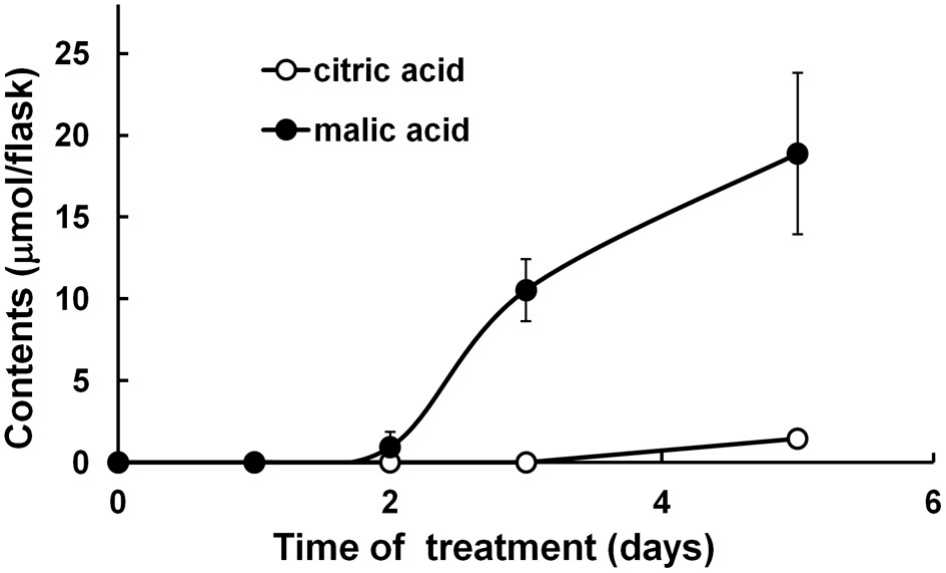
**Citric and malic acids released into the culture medium by *H. albus* roots under Fe-deficient conditions.** After *H. albus* root cultures had been exposed to Fe-deficient culture medium, 500 μL of the medium was collected at days 1, 2, 3, and 5, respectively. Results are the means of three independent experiments, and bars indicate standard deviations of the means.

In comparison to such proteins increased in abundance, a mitochondrial NAD-dependent malate dehydrogenase (MDH, spot 55) was decreased in relative intensity under Fe depletion (Table 3). It is interesting to note that an accumulation of large amounts of malate together with relatively small amounts of citrate was detected in the culture medium of *H. albus* roots (Figure 3). This is similar to the behavior of some other “strategy I” plant roots (Brown and Tiffin, 1965; Alhendawi et al., 1997; Abadía et al., 2002; Zocchi, 2006; Rellán-Alvarez et al., 2010) subjected to Fe-limiting conditions. The observed increase of fumarase (spot 41) could also contribute to malate secretion.

#### The defense system working under Fe deficiency

Our proteomic results revealed that the accumulations of predicted cationic peroxidase (spot 38) and ascorbate peroxidase (APX, spot 71) were decreased under Fe deficiency, while glutathione peroxidase (GPX, spot 61), CuZn-superoxide dismutase (SOD, spot 15), peroxidase 27 putative (spot 1), peroxiredoxin (PRX, spot 62) and pyridoxine biosynthesis protein isoform A (spot 7) all showed increased accumulation (Tables 2, 3). Generally, environmental stress enhances the production of reactive oxygen species (ROS) in plant cells, which can cause oxidative damage to lipids, proteins, and DNA, thereby promoting cell damage. Since the detoxification of ROS is essential for survival, many antioxidant enzymes (e.g., catalase, APX, SOD, GPX, and PRX) are involved in ROS-scavenging. Recently, pyridoxine biosynthesis protein is also revealed to be an efficient quencher of various ROS such as singlet oxygen and superoxide (Ehrenshaft et al., 1999; Chen and Xiong, 2005; Ristilä et al., 2011).

Our results suggest that increased accumulation of the Fe-independent enzymes, GPX, PRX, CuZnSOD and pyridoxine biosynthesis protein isoform A, may compensate for a decreased accumulation of the heme-containing ROS scavengers such as peroxidases and APX. On the other hand, the role of the newly detected putative peroxidase 27 (spot 1), a member of the class of pathogenesis-related proteins (Okushima et al., 2000), is not clear at present, because very little is known about this protein. It might play a crucial role in iron homeostasis under Fe deficiency.

#### Amino acid and protein metabolism under Fe deficiency

Two protease enzymes, cysteine protease 14 (spot 17) and aspartic protease (spot 60), were increased under Fe-deficient conditions (Table 2). Conversely, nitrite reductase (spot 44), responsible for assimilation of NO^−^_2_ (Takahashi et al., 2001) was found to be decreased by Fe deficiency (Table 3). In addition, decrease was observed of *S*-adenosyl-L-methionine synthetase (SAM, spots 69 and 70), which catalyzes the biosynthesis of SAM from methionine and ATP, and H6H (spot 30), which is involved in tropane alkaloid biosynthesis (Hashimoto and Yamada, 1987) (Table 3). Our proteomic data therefore suggested the possibility of a shift under Fe-deficient conditions away from the *de novo* synthesis of amino acids and toward an enhanced reutilization of amino acids produced by proteolysis. There might also have been some shift in the utilization of amino acids, toward essential cellular housekeeping and away from non-essential functions such as secondary product biosynthesis. It is well known that tropane alkaloids are derived from ornithine and phenylalanine. In *Cucumis sativus* plants, the assay of the nitrate reductase activity revealed the reduction of nitrate assimilation in both roots and leaves under Fe limitation (Borlotti et al., 2012). Based on this and the previous results, the authors hypothesized that the decreased proteins, actin, tubulin and globulin in this case, might be recycled and used as a source of amino acids, carbon skeletons and N-NH^+^_4_ under Fe deficiency (Donnini et al., 2010; Borlotti et al., 2012). Although the proteins detected in *H. albus* roots under Fe limitation were different from those in *C. sativus* roots, similar outline of an adaptation mechanism has been suggested from our results, too.

Nitrite reductase contains Fe-S clusters (Taiz and Zeiger, 2002), SAM synthetase requires ATP, as mentioned above, and H6H is a Fe-dependent enzyme. All these enzymes appeared from our proteomic data to be decreased in response to Fe deficiency, in agreement with our hypothesis that plants can survive under Fe deficiency by prioritizing the use of Fe and ATP for essential processes.

#### Fe deficiency induces morphological changes

In *H. albus* roots, ubiquitin-conjugating enzyme (UBC) E2 variant 13 (synonymous with UBC13) (spot 21) showed an increase in abundance under Fe deficiency (Table 2). In addition, actin (spot 59) and actin depolymerizing factor 3 (ADF, spot 53) were both increased under Fe deficiency (Table 2). Furthermore, an increase of galactose oxidase/kelch repeat-containing protein (Glx, spot 5), which is a member of the Cu-dependent Glx family of proteins involved in the oxidation of primary alcohols to aldehydes, was observed (Table 2). In contrast, one annexins p34 (spot 47), members of a multi-gene family, was decreased under Fe deficiency (Table 3). *Arabidopsis* responds to Fe deficiency by forming branched roots, and over-expression of UBC13 enhances this process (Li and Schmidt, 2010). In fact, *H. albus* roots showed morphological changes such as swelling of the root tips and increases in the numbers of lateral roots under Fe deficiency (Higa et al., 2008), same as previous report (Ling et al., 2002). An increase of ADF was also found in *Arabidopsis* (Lan et al., 2011). Changes in the actin cytoskeleton may be involved in adaptations to stresses, beginning with signal transduction and perception (Samaj et al., 2004). This possibility is supported by the fact that ADFs function in the remodeling of the actin cytoskeleton in response to environmental cues (Bamburg, 1999; Ruzicka et al., 2007). One annexins p34 (spot 47), which is capable of binding to F-actin (Hoshino et al., 2004) and of hydrolyzing ATP and GTP (Shin and Brown, 1999), was decreased under Fe deficiency (Table 3), possibly because of diminished availability of ATP or GTP.

One of the remarkable differences between our study and other proteomic studies of responses to Fe deficiency was the presence and enhanced expression of Glx (spot 5) (Table 2). However, the physiological role of this protein remains elusive. Recently, Liman et al. (2013) reported that Glx is required for aerial hyphae formation and for their further differentiation into hyphal spores in *Streptomyces coelicolor*. Similarly, this protein might play an important role in plant cell differentiation or in morphological changes.

#### Fe deficiency affects secondary metabolism and others

A major secondary metabolic pathway in *H. albus* is tropane alkaloid biosynthesis. The decrease of H6H in response to Fe deficiency has already been described above. In addition, two iso-forms of caffeoyl-CoA-*O*-methyltransferase (1 and 6: spots 23 and 18, respectively) involved in phenylpropanoid biosynthesis were increased under Fe deficiency (Table 2). This enzyme catalyzes the conversion of caffeoyl-CoA to feruloyl-CoA. On the other hand, sinapyl alcohol dehydrogenase (spot 66), involved in lignin biosynthesis and known to generate free-radical intermediates (Taiz and Zeiger, 2002), was found to decrease (Table 3). These results suggest that the phenylpropanoid pathway leading to lignin biosynthesis via sinapyl alcohol could be rerouted to produce other phenolics such as the coumarin derivative, scopoletin, found in *Arabidopsis* roots under Fe deficiency (Lan et al., 2011). However, in the case of *Arabidopsis*, increased lignification was suggested, because of the up-regulation of various enzymes, including PAL, involved in lignin biosynthesis (Lan et al., 2011; Rodríguez-Celma et al., 2013b). Lignifications usually occur after cell wall expansion ceases and synthesis of secondary cell wall starts (Taiz and Zeiger, 2002). Since under Fe deficiency, *H. albus* root tips start changing morphologically such as swelling, it is likely that liginification is suppressed under this situation. To confirm such metabolic changes in *H. albus* roots, further work is necessary.

One increased protein, 6,7-dimethyl-8-ribityllumazine synthase (RibC, spot 24) that we identified was associated with riboflavin production (Table 2). We will describe about this in the section Effects of Iron Deficiency on Tropane Alkaloid and Riboflavin Biosyntheses. Other increased and decreased proteins include soluble inorganic pyrophosphatase (spot 49) and acetoacetyl-CoA thiolase (spot 52), respectively (Tables 2, 3). Inorganic pyrophosphatase catalyzes the conversion of one molecule of pyrophosphate to two phosphate ions. This is a highly exergonic reaction and involved in many biochemical reactions, including lipid degradation and glycolysis (Jelitto et al., 1992). Acetoacetyl-CoA thiolases catalyze a reversible Claisen-type condensation of two acetyl-CoA molecules to form acetoacetyl-CoA and the first step of the mevalonate pathway (Ahumada et al., 2008). These changes are likely to be involved in the survival strategy of *H. albus*.

#### Changes in ETC and ATP synthesis by Fe deficiency

It was found that a component of the mitochondrial ETC, complex I, NADH dehydrogenase Fe-S protein 1 (spot 27), which requires many Fe ions (Ohnishi, 1998), was decreased in abundance (Table 3). An abundance decrease of ATP synthase subunit alpha (spot 39) was also observed (Table 3). Decreased acuumulation of a subunit of complex I under Fe deficiency has also been reported in *M. truncatula* (Rodríguez-Celma et al., 2011a). This result was consistent with our own previous results obtained through inhibitor-feeding experiments (Higa et al., 2010), and with the results of enzyme assay and western analysis in cucumber roots (Vigani et al., 2009).

As mentioned previously, our proteomic data showed that most of the proteins that were decreased required either Fe or ATP for activity. This is also applied to complex I as well. In addition, the observed decrease of ATP synthase subunit alpha (spot 39) suggested a reduced capacity for ATP synthesis as well.

### Effects of iron deficiency on tropane alkaloid and riboflavin biosyntheses

**Figure 4:**
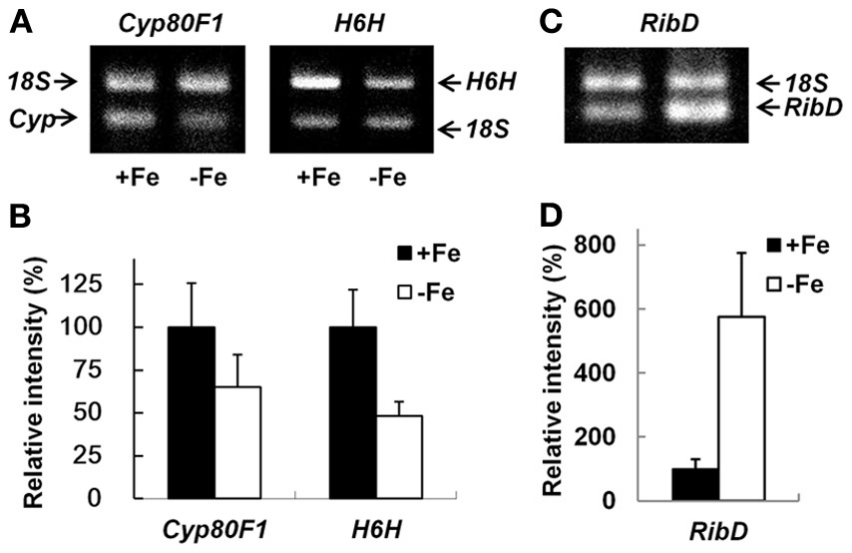
**Transcript accumulations of *H6H, Cyp80F1* and *RibD* in the root tips of *H. albus* roots cultured under Fe-deficient and Fe-replete conditions.** Representative expression profiles are shown in **(A)** and **(C)** and semiquantitative analysis of the expressions with three biological replicates and standard deviations are in **(B)** and **(D)**, respectively. H6H, hyoscyamine 6β-hydoroxylase, catalyzes the conversion from hyoscyamine to scopolamine, Cyp80F1, a cytochrome P450, is involved in littorine rearrangement to produce hyoscyamine and RibD, riboflavin synthase, is involved in *de nono* synthesis of ribofalvin. RNA was extracted from 0.1 g of fresh root tips, and 500–1000 ng RNA was used for RT-PCR. Using primers specific for *H6H, Cyp80F1* and *RibD*, corresponding fragments of 512 bp, 202 bp and 215 bp, respectively, were amplified. Ribosomal RNA 18S universal primer pairs were used as an internal standard for normalization (product fragment, 315 bp). −Fe, no addition of Fe; +Fe, with addition of 0.1 mM Fe(III)-EDTA.

Because of the importance of *H. albus* as a source of medicinal compounds, we investigated further the effects of Fe deficiency on tropane alkaloid production. H6H (EC1.14.11.11), required Fe as a cofactor for activity, is a bi-functional monooxygenase and converts hyoscyamine to 6β-hydroxyhyoscyamine and thence to scopolamine (Hashimoto et al., 1993). In addition, hyoscyamine biosynthesis via the rearrangement of littorine is found to be catalyzed by a Fe-dependent cytochrome P450, Cyp80F1 (Li et al., 2006). Therefore, we decided to investigate whether these Fe-dependent enzymes were down-regulated at the expression level and whether tropane alkaloid content was decreased. The expression of both *Cyp80F1* and *H6H* was examined by RT-PCR, using hairy roots treated with/without Fe for 5 days (Figure 4). The results showed that the transcript accumulations of both enzymes in Fe-deficient root tips were reduced in comparison to those in Fe-replete root tips (Figure 4A). Semi-quantitative analysis showed that H6H (*ca*. 50% diminished) was more strongly affected than Cyp80F1 (*ca*. 35% diminished) (Figure 4B).

**Figure 5:**
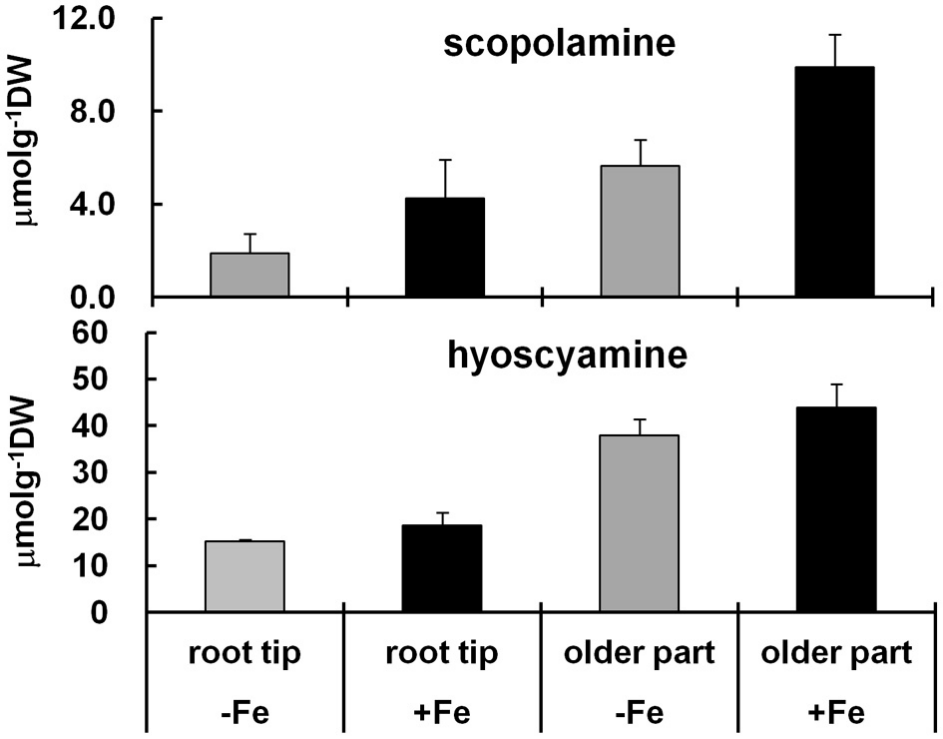
**Alkaloid contents of the tips and of the remaining older parts of *H. albus* roots cultured under Fe deficiency and Fe repletion.** Roots were treated with and without Fe for 5 days, and then harvested. Roots were separated into the tips (*ca*. 1.0–1.5 cm in length) and the remaining parts, and then dried at 50°C overnight. Results are means of three independent experiments, and bars indicate standard deviations of the means.

The effect of Fe deficiency on alkaloid production was also examined by HPLC, using not only root tips but also the older parts of the roots, because it is known that alkaloids can be transported *via* the xylem (Manske and Holmes, 1965). The results showed that the root tips accumulated smaller amounts of scopolamine and hyoscyamine than the older parts, and that the hyoscyamine content was always substantial, regardless of the root material (Figure 5). The results confirmed that the content of both hyoscyamine and scopolamine was decreased under Fe deficiency, but that the scopolamine content was more strongly affected than hyoscyamine content (Figure 5), in agreement with the results for transcript accumulation (Figures 4A,B). This might reflect the known requirement for Fe ions in the conversion of hyoscyamine to scopolamine.

Flavins are heterocyclic compounds same as alkaloids. In contrast to tropane alkaloid biosynthesis described above, accumulation of RibC that is involved in riboflavin synthsis was increased in the root tips treated without Fe for 5 days (Table 2). This result agreed with our previous findings that the riboflavin secretion we observed occurred as a result of the enhancement both of *de novo* riboflavin synthesis and of the hydrolysis of FMN (Higa et al., 2012), although protein that functions in the hydrolysis of FMN was not identified. To reconfirm the previous results, we again determined the gene expression of *riboflavin synthase* (*RibD*) as a direct enzyme involved in *de novo* riboflavin synthesis, by the same way used for analyses of *Cyp80F1* and *H6H* (Figure 4C). The semi-quantitative analysis showed an apparent increase (*ca*. 6 times) in *HaRibD* accumulation under Fe deficiency (Figure 4D). This coincided with the previous reports on both *M. truncatula* and *B. vulgaris*, in which increase of the riboflavin-biosynthetic enzyme, RibC was confirmed by proteomic data as well as by analysis at the mRNA level (Rellán-Alvarez et al., 2010; Rodríguez-Celma et al., 2011a).

In this paper, we reported the application of a small-scale proteomic approach that permitted the extraction and separation of proteins from 100 mg (fresh weight) of *H. albus* root tips. Two-dimensional separation using mini-gels, followed by spot-picking and analysis by Matrix-assisted laser desorption/ionization–quadrupole ion trap–time of flight (MALDI-QIT-TOF) mass spectrometry allowed the identification of 36 differentially accumulated proteins in Fe-deficient roots compared to Fe-replete roots. To the best of our knowledge, this is the first reported application of the use of MALDI-QIT-TOF mass spectrometry for the analysis of proteins from plant roots in relation to Fe deficiency. The proteome profile supported our hypothesis that the adaptation of *H. albus* roots under Fe depletion is characterized by metabolic and respiratory shifts that achieve greater economy in the use of Fe, one example of which was the suppression of tropane alkaloid biosynthesis.

One of the suggestions emerging from our proteomic data is that malate secretion seemed to occur as a result of a decreased abundance of mitochondrial NAD-dependent MDH under Fe deficiency (Table 3). Although the secretion of both malate and citrate is a well-known phenomenon, the mechanism of origin of malate remains uncertain. It has been suggested that the observed up-regulation of cytosolic phosphoenolpyruvate carboxylase (PEPC) (López-Millán et al., 2000) generates oxaloacetate and malate, which can be imported into mitochondria and thence generate citrate; citrate might then efflux from the mitochondria to be transported in the xylem and/or exuded (Zocchi, 2006). In the case of *H. albus* roots, it is uncertain whether malate that is secreted is derived from the mitochondria and/or the cytosol. In any event, an apparent decrease of mtMDH must cause metabolic changes in the mitochondria. Recently, in the case of breakdown of the TCA cycle, non-cyclic flux models have been proposed (Sweetlove et al., 2010; Vigani, 2012). The TCA cycle provides not only organic acids, but also amino acid precursors. Our proteomic data indicate, however, that under Fe deficiency amino acids for cell development are generated mainly by proteolysis, with the result that *de novo* synthesis may be reduced. This suggests that the TCA cycle may not be fully working under Fe deficiency.

Secreted citrate and malate have been suggested to function as carriers of Fe ions and as cytosolic pH stats after H^+^ efflux by H^+^-ATPase (Zocchi, 2006). However, for plants and from the perspective of energy consumption, the secretion of malate (a C_4_ compound) is more economical than that of citrate (a C_6_ compound). Since a flux of carbon from plants to the soil increases both the size of the microbial population and the mobilization of soil micronutrients (Dakora and Phillips, 2002), malate might in principle be used to invite fungi or bacteria as “vehicles” for the supply of Fe ions for plant roots. In addition to citrate and malate, flavin (riboflavin) secretion also occurred in the culture media of *H. albus* roots under Fe deficiency (Higa et al., 2008, 2010, 2012), same as *B. vulgaris* (Susin et al., 1994), *M. truncatula* (Rodríguez-Celma et al., 2011a,b), and *C. sativus* (Shinmachi et al., 1997). Together with organic acids or independently, flavins may aid the availability of soil Fe for plant roots *via* changing the soil microflora as indicated by Vorwieger et al. (2007). They suggested that riboflavin acts as a plant-generated signal to manipulate rhizosphere microbiology. Recent evidence that chemical diversity exists among secreted/accumulated flavins depending on plant species (Rodríguez-Celma et al., 2011b) also supports their ecological role. Another possible role of flavins produced by *M. truncatula* roots in the rhizosphere has been proposed, according to the result on the coexpression and promoter analysis of genes that are responsive to Fe deficiency: as iron-binding compounds, flavins chelate Fe(III) from non-soluble ferrihydroxides and provide these chelates to Fe reductase (Rodríguez-Celma et al., 2013b). The authors also mentioned that in the case of *Arabidopsis*, phenylpropanoids function as iron-binding compounds, and flavins and phenylpropanoids are produced by a mutually exclusive metabolism in these plants.

Further extensive studies are clearly required, however, in order to elucidate the ecological significance of organic acid and flavin secretions into the rhizosphere.

## Conclusion

*Hyoscyamus albus* is an important medicinal plant, as a source of both hyoscyamine and scopolamine. Using our original small-scale proteomic approach with 100 mg of root tips, we determined the effects of Fe deficiency on protein expression. The differentially-accumulated proteins (36) identified by MALDI-QIT-TOF mass spectrometry were divided into six groups according to function, *viz*: carbohydrate metabolism, defense responses, amino acid/protein metabolism, structure/development, ETC /ATP synthesis and, lastly, other miscellaneous functions, including secondary metabolism.

There was evidence of the enhanced accumulation of some glycolytic proteins under Fe-deficient conditions, presumably to maintain energy supply, but the decrease of mitochondrial MDH under Fe deficiency seemed correlated with malate secretion into the rhizosphere. For defense against environmental stresses, there was evidence that Fe-independent ROS scavenging machinery might substitute for Fe-dependent mechanisms. Our data also confirmed that the response to Fe deficiency caused morphological changes such as root branching. Furthermore, the proteins necessary for such remodeling of root development seemed to derive their amino acids through proteolysis, rather than through amino acid biosynthesis *de novo*. Fe deficiency also affected secondary metabolism, including alkaloid metabolism. Most proteins exhibiting decreases in abundance required either Fe or ATP for activity. Representative examples were the mitochondrial ETC component complex I and H6H, which is involved in tropane alkaloid biosynthesis. A further determination of transcript accumulation for H6H supported the decrease in tropane alkaloid content observed in Fe-deficient roots. On the contrary, the increase of RibC accumulation indicated that riboflavin secretion occurs at least through the enhancement of *de novo* synthesis.

Economy in the use of Fe and ATP, including the use of energy to re-programme root morphology and function in response to Fe restriction, must be a principal strategy for plant roots faced with severely suboptimal Fe availability. In contrast, another strategy of expenditure of carbon and nitrogen sources in the rhizosphere need to be solved.

### Conflict of interest statement

The authors declare that the research was conducted in the absence of any commercial or financial relationships that could be construed as a potential conflict of interest.
