# Supplementary material for: A small-scale proteomic approach reveals a survival strategy, including a reduction in alkaloid biosynthesis, in Hyoscyamus albus roots subjected to iron deficiency
Source: Front Plant Sci. 2013 Aug 28;4:331. doi: 10.3389/fpls.2013.00331 (PMC3755260; doi:10.3389/fpls.2013.00331)
Supplement: Supplementary file 1 [file DataSheet1.PDF]

Supplementary data 1. Results of MS/MS ion search and BLAST search for protein identification

Spot No[a]	Protein ID	EST (Accession No.)[b]	Homolog (Accession No.)[c]	Species[d]	m/z	Sequence	Delta	Miss[e]	Score	Expect
**Carbohydrate Metabolism**										
55	NAD-dependent malate dehydrogenase	gi| 190836589	CAB45387.1	*Nicotiana tabacum*	1348.00	KLFGVTTLDVVR	0.20	1	39	5.2E-01
					2081.41	TKPSTTFTDEEVQELTVR	0.37	0	31	1.9E+00
40	Fructose bisphosphate aldolase-like protein	gi|224620481	ABC01905.1	*Solanum tuberosum*	1346.91	VAPEVIAEYTVR	0.18	0	23	1.8E+01
					2052.31	IGANEPSQLAINENANGLAR	0.26	0	42	1.5E-01
58	UDP-glucose pyrophosphorylase	ND	UGPA_SOLTU	*Solanum tuberosum*	1744.12	SNPSNPSIELGPEFKK	0.23	1	51	7.3E-03
28	Phosphoglycerate mutase	gi|261472980	AAD24857.1	*Solanum tuberosum*	1188.82	YLVSPPEIDR	0.19	0	25	1.5E+01
					2079.38	IHYAGMLQYDGELKLPSK	0.32	1	24	1.0E+01
					2094.48	YLVSPPEIDRTSGEYLVR	0.39	1	17	4.3E+01
29	Phosphoglycerate mutase	gi|261472980	XM_004243571.1	*Solanum lycopersicum*	1188.80	YLVSPPEIDR	0.17	0	26	9.6E+00
					2094.38	YLVSPPEIDRTSGEYLVR	0.30	1	21	1.2E+01
22	Enolase	gi|225923250	NM_001247151.2	*Solanum lycopersicum*	1827.87	IEEELGSEAVYAGASFR	-0.01	0	56	7.1E-03
					1844.90	VVIGMDVAASEFYGKDK	-0.01	1	18	3.9E+01
32	Fructose bisphosphate aldolase-like protein	gi|254603758	ABB29926.1	*Solanum tuberosum*	2052.93	IGANEPSQLAINENADGLAR	-0.10	0	21	1.7E+01
					2316.13	GTVELPGTNGETTTQGLDGLAE	0.00	0	31	1.6E+00
41	Fumarase	gi|190858454	CAA62817.1	*Solanum tuberosum*	1078.93	SVEFKDIIK	0.32	1	56	1.1E-02
**Defense Response**										
71	Ascorbate peroxidase	gi|190791061	BAA12918.1	*Nicotiana tabacum*	1556.18	ALLSDPAFRPLVEK	0.30	0	16	8.4E+01
					1595.07	LAWHSAGTYDVCSK	0.34	0	61	2.5E-03
					1602.14	GLIAEKNCAPLMLR	0.29	1	10	3.3E+02
					1636.13	AEQGHGANNGIDIAIR	0.31	0	13	1.6E+02
					1911.35	FKAEQGHGANNGIDIAIR	0.37	1	46	5.8E-02
					2047.31	YAADEDAFFADYAEAHLK	0.40	0	56	5.7E-03
38	Predicted cationic peroxidase	gi|227581441	XP_002268412.2	*Vitis vinifera*	1419.92	TFSKDFAESMIK	0.24	1	48	5.5E-02
7	Pyridoxine biosynthesis protein isoform A	gi|190788880	XP_004137720.1	*Cucumis melo*	1474.33	GGVIMDVVNAEQAR	-0.40	0	18	5.7E+01
					1515.38	TKGEAGTGNIIEAVR	-0.43	1	39	4.8E-01
9	Chaperonin 21 precursor	gi|224617172	NP_001234423.1	*Solanum lycopersicum*	1149.42	YTSLKPLGDR	-0.21	0	15	1.3E+02
					1302.36	YAGTEVEFDGSK	-0.22	0	48	6.5E-02
1	Peroxidase 27 putative	gi|227578805	XP_002280216.1	*Vitis vinifera*	1640.98	GNQAEKDAIPNQSLR	0.14	1	52	1.9E-02
62	Peroxiredoxin	gi|326309231	AAP42502.1	*Ipomoea batatas*	1747.90	DAIEFYGDFDGSFHK	0.14	0	28	4.1E+00
					2188.23	LQAKDAIEFYGDFDGSFHK	0.20	1	41	1.8E-01

					2386.42	VLNVEEAPSDFQVSGGDVILGQ	0.21	0	25	5.9E+00
61	Glutathione peroxidase	gi|224614384	CAJ00224.1	*Capsicum chinense*	1199.53	SSKGGFFGDGIK	-0.08	1	23	2.2E+01
					1257.55	FKAEYPIFDK	-0.10	1	14	1.8E+02
					1559.64	GGFFGDGIKWNFSK	-0.12	1	52	2.0E-02
					1575.64	GGFFGDGIKWNFSK	-0.12	1	26	7.7E+00
15	Superoxide dismutase [Cu-Zn]	gi|309375176	P27082.2	*Nicotiana plumbaginifolia*	1829.29	AVVVHADPDDLGKGGHEL	0.37	1	50	2.8E-02
					2319.60	HAGDLGNITVGEDGTASFTITD	0.49	0	51	1.7E-02
**Structure / Development**										
47	Annexin p34	gi|283774589	NP_001234104.1	*Solanum lycopersicum*	1562.83	QLEDDDEFVALLR	0.06	0	10	3.6E+02
					1692.90	GLAYPEHYFVEVLR	0.03	0	39	3.9E-01
59	Actin	ND	ACTA_BOVIN	*Bos taurus*	1790.96	SYELPDGQVITIGNER	0.07	0	29	1.3E+00
					1956.13	VAPEEHPTLLTEAPLNPK	0.09	0	30	9.0E-01
21	Ubiquitin-conjugating enzyme E2 variant 13	gi| 227581295	ABA40444.1	*Solanum tuberosum*	1278.76	KLVQPPEGTYF	0.09	1	23	1.9E+01
					1847.99	SWTGTIIGPHNSVHEGR	0.08	0	11	2.0E+02
					1865.02	LFCDKDYPEKPPTVR	0.09	1	44	1.1E-01
53	Actin depolymerizing factor 3	gi|220676147	ABD66505.1	*Gossypium hirsutum*	1152.77	FIVFKIEEK	0.10	1	48	5.8E-02
					1381.80	YAVYDFDFLTK	0.13	0	26	1.1E+01
5	Galactose oxidase/kelch repeat-containing protein	gi|190795012	EFH58804.1	*Arabidopsis lyrata*	1284.71	IYIFGGCGNAGR	0.09	0	21	2.9E+01
					1440.88	LGGIFFFTPLVET	0.10	0	30	3.9E+00
**Amino Acid / Protein Metabolism**										
46	Elongation factor Tu	ND	EFTM_ARATH	*Arabidopsis thaliana*	1341.14	KFEAEIYVLTK	0.39	1	62	2.7E-03
					1576.26	AIAFDEIDKAPEEK	0.47	1	45	3.40E-02
					1614.35	TADITGKVELPENVK	0.48	1	30	3.4E+00
					1649.28	LMDAVDEYIPDPVR	0.49	0	33	1.8E+00
					1668.4	ILDNGQAGDNVGLLLR	0.49	0	21	7.50E+00
70	*S*-adenosylmethionine synthase	ND	METKI_BRARP	*Brassica rapa*	1776.01	YLDENTIFHLNPSGR	0.47	1	23	5.6E+00
					1454.16	FVIGGPHGDAGLTGR	0.41	0	27	2.0E+00
69	*S*-adenosylmethionine synthase 2	ND	METK2_SOLTU	*Solanum tuberosum*	1776.01	YLDENTIFHLNPSGR	0.14	0	23	4.7E+00
					2313.06	TAAYGHFGRDDPDFTWETVK	0.00	1	29	1.2E+00
44	Nitrite reductase	gi|283773379	BAD15364.1	*Nicotiana tabacum*	1192.65	TVEGADVFLGGK	0.03	0	39	5.3E-01
					1589.81	NFGAVPREIEEAEE	0.07	1	18	5.6E+01
60	Aspartic protease	gi|326311224	ABG37021.1	*Nicotiana tabacum*	1680.01	FDGILGLGFQEISVGK	0.11	0	31	2.5E+00
					1876.00	NAEEEEGGELVFGGVDPK	0.14	0	37	5.2E-01
17	Cysteine protease 14	gi|224679931	AAP32193.1	*Trifolium repens*	1640.83	ALANQPLSVAIEASTR	0.40	0	49	4.0E-02

**Secondary Metabolism / Others**										
66	Sinapyl alcohol dehydrogenase	gi|283772972	XP_002322822.1	*Populus trichocarpa*	1673.17	FVIDVENTLVAAEPR	0.28	0	28	5.4E+00
					1760.21	FVIDVENTLVAAEPRS	0.29	1	40	2.9E-01
30	Hyoscyamine 6β-hydroxylase	gi|283774237	AAT40509.2	*Solanum demissum*	1137.62	SFSYTEYLK	0.07	0	12	2.7E+02
					1409.86	VISNGKLESGIHR	0.07	1	17	7.4E+01
					1447.85	ALLSESTNPPKYK	0.07	1	19	4.6E+01
					2106.15	IFFTDTSEFEAALNPYKI	0.11	1	15	6.1E+01
52	Acetoacetyl-CoA	gi|190786416	AAU95618.1	*Nicotiana tabacum*	1616.00	EEQDDYAVQSFER	0.31	0	18	5.3E+01
					1986.26	ITREEQDDYAVQSFER	0.34	1	32	1.4E+00
18	Caffeoyl-CoA-O-methyltransferase 6	gi|190787138	XP_002313125.1	*Nicotiana tabacum*	1459.93	YYRDFVLELNK	0.17	1	59	7.3E-03
23	Caffeoyl-CoA-O-methyltransferase 1	ND	CAMT1_TOBAC	*Nicotiana tabacum*	1113.73	AGLAHKIEFK	0.09	1	39	1.5E-01
					1459.90	YYRDFVLELNK	0.14	1	40	1.2E-01
49	Soluble inorganic pyrophosphatase	gi|224690623	Q43187.1	*Solanum tuberosum*	2016.29	ILYSSVVYPQNYGFIPR	0.23	0	58	1.4E-03
24	6,7-Dimethyl-8-ribityllumazine synthase	gi|397174509	BAM34417	*Hyoscyamus albus*	1721.19	SQKYEAILCIGAVIR	0.25	1	55	8.80E-03
**ETC/ATP Synthesis**										
27	NADH dehydrogenase Fe-S protein 1	ND	NDUS1_SOLTU	*Solanum tuberosum*	1667.04	FASEVAGVEDLGMLGR	0.23	0	19	1.2E+01
					1888.27	ALSEVAGVGLPYDSLGAIR	0.25	0	26	2.5E+00
39	ATP synthase subunit alpha	gi|227580017	ADL63175.1	*Celtis yunnanensis*	1538.00	EAFPGDVFYLHSR	0.26	0	41	2.1E-01
					1721.07	DNGMHALIIYDDLSK	0.25	0	21	2.1E+01

[a] Spot number that corresponds to Prodigy rank number.

[b] Accession number of the top hit sequence from the Solanaceae EST database except 6, 7-Dimethyl-8-ribityllumazine synthase from NCBInr is shown.
ND: Not determined, as a homolog was hit from SwissProt.

[c] Accession number of the top hit homolog from MS/MS ion search (MIS) using SwissProt database or MIS followed by EST-based BLAST search using non-redundant protein d

[d] Source organism of the 'homolog' indicated in the left column.

[e] Number of missed cleavage sites in the tryptic fragment.
