# Supplementary material for: A small-scale proteomic approach reveals a survival strategy, including a reduction in alkaloid biosynthesis, in Hyoscyamus albus roots subjected to iron deficiency
Source: Front Plant Sci. 2013 Aug 28;4:331. doi: 10.3389/fpls.2013.00331 (PMC3755260; doi:10.3389/fpls.2013.00331)

Spot 1. Peroxidase 27 putative

Peptide View

MS/MS Fragmentation of **GNQAEKDAIPNQSLR**
Found in **gi|227578805**, FN004358 normalized cDNA library of roots from Petunia x hybrida, Mitchell diploid (W115) Petunia x hybrida cDNA clone dr001P0013I10_F.ab1 2007-08-10, mRNA sequence
Translated in frame 1 (nucleic acid sequence)

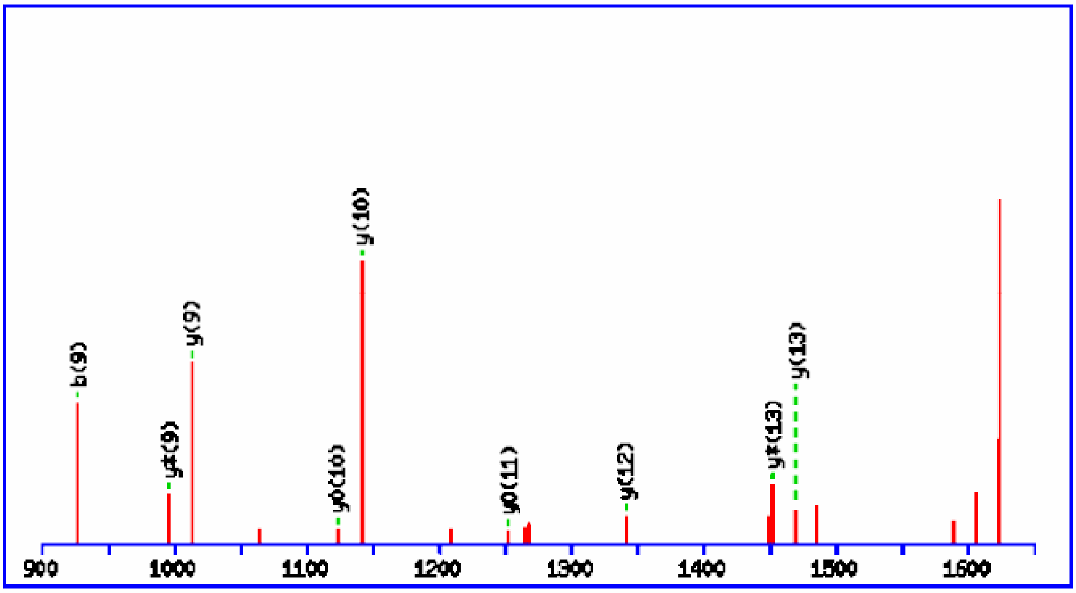


```
Monoisotopic mass of neutral peptide Mr(calc): 1639.8278
Fixed modifications: Carbamidomethyl (C) (apply to specified residues or termini only)
Ions Score: 52  Expect: 0.019
Matches : 29/232 fragment ions using 39 most intense peaks   (help)
```

#	Immon.	a	a*	a^0	b	b*	b^0	Seq.	y	y*	y^0	#
1	30.0338	30.0338			58.0287			G				15
2	87.0553	144.0768	127.0502		172.0717	155.0451		N	1583.8136	1566.7871	1565.8030	14
3	101.0709	272.1353	255.1088		300.1302	283.1037		Q	1469.7707	1452.7441	1451.7601	13
4	44.0495	343.1724	326.1459		371.1674	354.1408		A	1341.7121	1324.6856	1323.7015	12
5	102.0550	472.2150	455.1885	454.2045	500.2100	483.1834	482.1994	E	1270.6750	1253.6484	1252.6644	11
6	101.1073	600.3100	583.2835	582.2994	628.3049	611.2784	610.2944	K	1141.6324	1124.6058	1123.6218	10
7	88.0393	715.3369	698.3104	697.3264	743.3319	726.3053	725.3213	D	1013.5374	996.5109	995.5269	9
8	44.0495	786.3741	769.3475	768.3635	814.3690	797.3424	796.3584	A	898.5105	881.4839	880.4999	8
9	86.0964	899.4581	882.4316	881.4476	927.4530	910.4265	909.4425	I	827.4734	810.4468	809.4628	7
10	70.0651	996.5109	979.4843	978.5003	1024.5058	1007.4793	1006.4952	P	714.3893	697.3628	696.3787	6
11	87.0553	1110.5538	1093.5273	1092.5432	1138.5487	1121.5222	1120.5382	N	617.3365	600.3100	599.3260	5
12	101.0709	1238.6124	1221.5858	1220.6018	1266.6073	1249.5808	1248.5967	Q	503.2936	486.2671	485.2831	4
13	60.0444	1325.6444	1308.6179	1307.6339	1353.6393	1336.6128	1335.6288	S	375.2350	358.2085	357.2245	3
14	86.0964	1438.7285	1421.7019	1420.7179	1466.7234	1449.6968	1448.7128	L	288.2030	271.1765		2
15	129.1135							R	175.1190	158.0924		1

Seq	ya	yb	Seq	ya	yb	Seq	ya	yb
NQ	215.1139	243.1088	NQA	286.1510	314.1459	NQAE	415.1936	443.1885
NQAEK	543.2885	571.2835	NQAEKD	658.3155	686.3104	QA	172.1081	200.1030
QAE	301.1506	329.1456	QAEK	429.2456	457.2405	QAEKD	544.2726	572.2675
QAEKDA	615.3097	643.3046	AE	173.0921	201.0870	AEK	301.1870	329.1819
AEKD	416.2140	444.2089	AEKDA	487.2511	515.2460	AEKDAI	600.3352	628.3301
AEKDAIP	697.3879	725.3828	EK	230.1499	258.1448	EKD	345.1769	373.1718
EKDA	416.2140	444.2089	EKDAI	529.2980	557.2930	EKDAIP	626.3508	654.3457
KD	216.1343	244.1292	KDA	287.1714	315.1663	KDAI	400.2554	428.2504
KDAIP	497.3082	525.3031	KDAIPN	611.3511	639.3461	DA	159.0764	187.0713
DAI	272.1605	300.1554	DAIP	369.2132	397.2082	DAIPN	483.2562	511.2511
DAIPNQ	611.3148	639.3097	DAIPNQS	698.3468	726.3417	AI	157.1335	185.1285
AIP	254.1863	282.1812	AIPN	368.2292	396.2241	AIPNQ	496.2878	524.2827
AIPNQS	583.3198	611.3148	AIPNQSL	696.4039	724.3988	IP	183.1492	211.1441
IPN	297.1921	325.1870	IPNQ	425.2507	453.2456	IPNQS	512.2827	540.2776
IPNQSL	625.3668	653.3617	PN	184.1081	212.1030	PNQ	312.1666	340.1615
PNQS	399.1987	427.1936	PNQSL	512.2827	540.2776	NQ	215.1139	243.1088
NQS	302.1459	330.1408	NQSL	415.2300	443.2249	QS	188.1030	216.0979
QSL	301.1870	329.1819	SL	173.1285	201.1234			

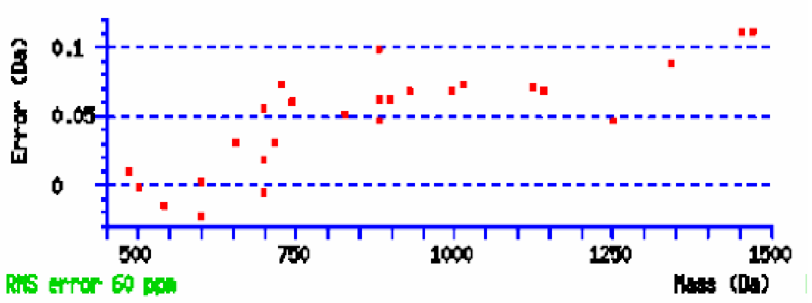

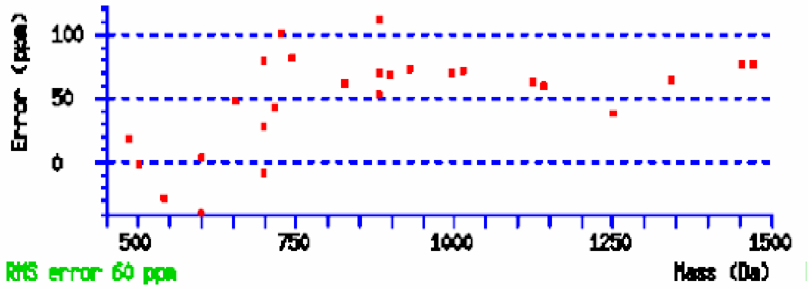

Spot 17. Cysteine protease 14

Peptide View

MS/MS Fragmentation of **ALANQPLSVAIEASTR**
Found in **gi|224679931**, KS25024N12 KS25 Capsicum annuum cDNA, mRNA sequence
Translated in frame 5 (nucleic acid sequence)

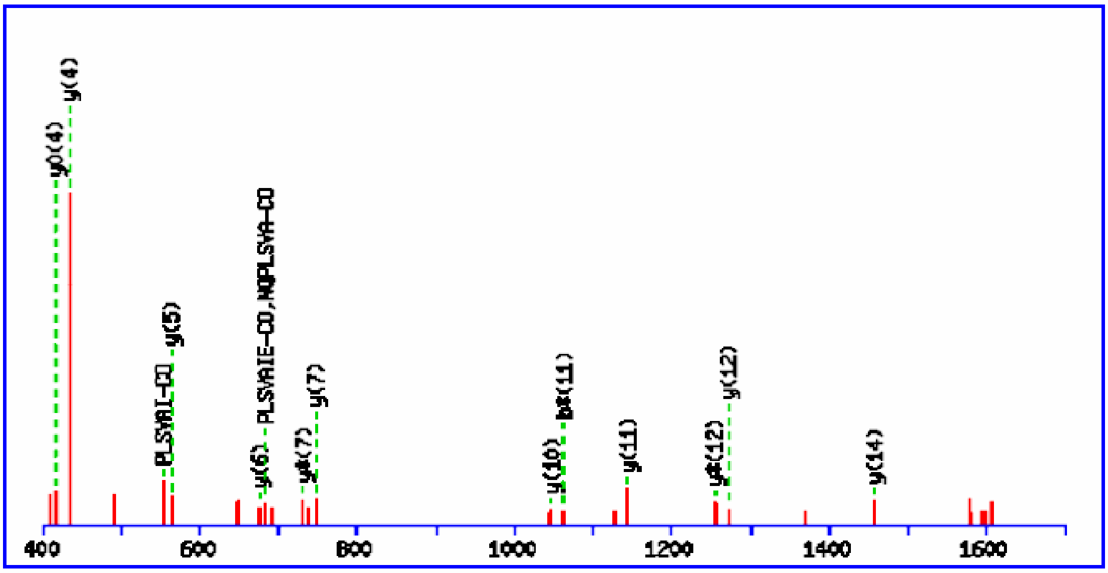


```
Monoisotopic mass of neutral peptide Mr(calc): 1639.8893
Ions Score: 49  Expect: 0.04
Matches : 16/251 fragment ions using 24 most intense peaks   (help)
```

#	Immon.	a	a*	a^0	b	b*	b^0	Seq.	y	y*	y^0	#
1	44.0495	44.0495			72.0444			A				16
2	86.0964	157.1335			185.1285			L	1569.8595	1552.8329	1551.8489	15
3	44.0495	228.1707			256.1656			A	1456.7754	1439.7489	1438.7649	14
4	87.0553	342.2136	325.1870		370.2085	353.1819		N	1385.7383	1368.7118	1367.7278	13
5	101.0709	470.2722	453.2456		498.2671	481.2405		Q	1271.6954	1254.6688	1253.6848	12
6	70.0651	567.3249	550.2984		595.3198	578.2933		P	1143.6368	1126.6103	1125.6262	11
7	86.0964	680.4090	663.3824		708.4039	691.3774		L	1046.5840	1029.5575	1028.5735	10
8	60.0444	767.4410	750.4145	749.4304	795.4359	778.4094	777.4254	S	933.5000	916.4734	915.4894	9
9	72.0808	866.5094	849.4829	848.4989	894.5043	877.4778	876.4938	V	846.4680	829.4414	828.4574	8
10	44.0495	937.5465	920.5200	919.5360	965.5415	948.5149	947.5309	A	747.3995	730.3730	729.3890	7
11	86.0964	1050.6306	1033.6041	1032.6200	1078.6255	1061.5990	1060.6150	I	676.3624	659.3359	658.3519	6
12	102.0550	1179.6732	1162.6466	1161.6626	1207.6681	1190.6416	1189.6575	E	563.2784	546.2518	545.2678	5
13	44.0495	1250.7103	1233.6838	1232.6997	1278.7052	1261.6787	1260.6947	A	434.2358	417.2092	416.2252	4
14	60.0444	1337.7423	1320.7158	1319.7318	1365.7373	1348.7107	1347.7267	S	363.1987	346.1721	345.1881	3
15	74.0600	1438.7900	1421.7635	1420.7795	1466.7849	1449.7584	1448.7744	T	276.1666	259.1401	258.1561	2
16	129.1135							R	175.1190	158.0924		1

Seq	ya	yb	Seq	ya	yb	Seq	ya	yb
LA	157.1335	185.1285	LAN	271.1765	299.1714	LANQ	399.2350	427.2300
LANQP	496.2878	524.2827	LANQPL	609.3719	637.3668	LANQPLS	696.4039	724.3988
AN	158.0924	186.0873	ANQ	286.1510	314.1459	ANQP	383.2037	411.1987
ANQPL	496.2878	524.2827	ANQPLS	583.3198	611.3148	ANQPLSV	682.3882	710.3832
NQ	215.1139	243.1088	NQP	312.1666	340.1615	NQPL	425.2507	453.2456
NQPLS	512.2827	540.2776	NQPLSV	611.3511	639.3461	NQPLSVA	682.3882	710.3832
QP	198.1237	226.1186	QPL	311.2078	339.2027	QPLS	398.2398	426.2347
QPLSV	497.3082	525.3031	QPLSVA	568.3453	596.3402	QPLSVAI	681.4294	709.4243
PL	183.1492	211.1441	PLS	270.1812	298.1761	PLSV	369.2496	397.2445
PLSVA	440.2867	468.2817	PLSVAI	553.3708	581.3657	PLSVAIE	682.4134	710.4083
LS	173.1285	201.1234	LSV	272.1969	300.1918	LSVA	343.2340	371.2289
LSVAI	456.3180	484.3130	LSVAIE	585.3606	613.3556	LSVAIEA	656.3978	684.3927
SV	159.1128	187.1077	SVA	230.1499	258.1448	SVAI	343.2340	371.2289
SVAIE	472.2766	500.2715	SVAIEA	543.3137	571.3086	SVAIEAS	630.3457	658.3406
VA	143.1179	171.1128	VAI	256.2020	284.1969	VAIE	385.2445	413.2395
VAIEA	456.2817	484.2766	VAIEAS	543.3137	571.3086	VAIEAST	644.3614	672.3563
AI	157.1335	185.1285	AIE	286.1761	314.1710	AIEA	357.2132	385.2082
AIEAS	444.2453	472.2402	AIEAST	545.2930	573.2879	IE	215.1390	243.1339
IEA	286.1761	314.1710	IEAS	373.2082	401.2031	IEAST	474.2558	502.2508
EA	173.0921	201.0870	EAS	260.1241	288.1190	EAST	361.1718	389.1667
AS	131.0815	159.0764	AST	232.1292	260.1241	ST	161.0921	189.0870

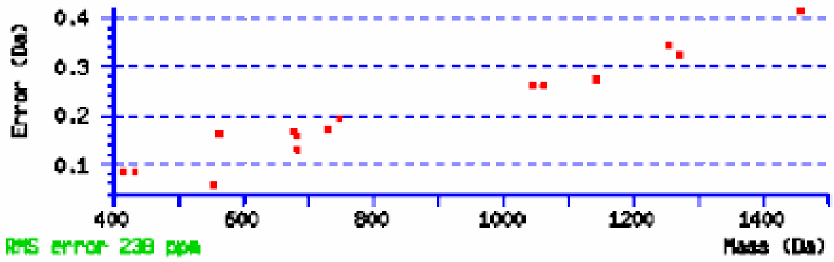

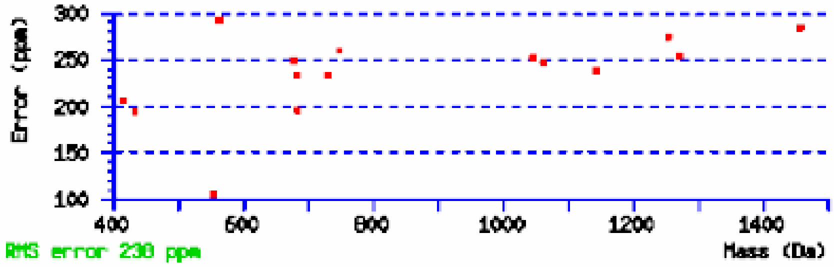

Spot 18. Caffeoyl-CoA-O-methyltransferase 6

Peptide View

MS/MS Fragmentation of **YYRDFVLELNK**
Found in **gi|190787138**, AGN_PNL220dr1_h4.trimmed.seq AGN_PNL Nicotiana tabacum cDNA 3', mRNA sequence
Translated in frame 6 (nucleic acid sequence)

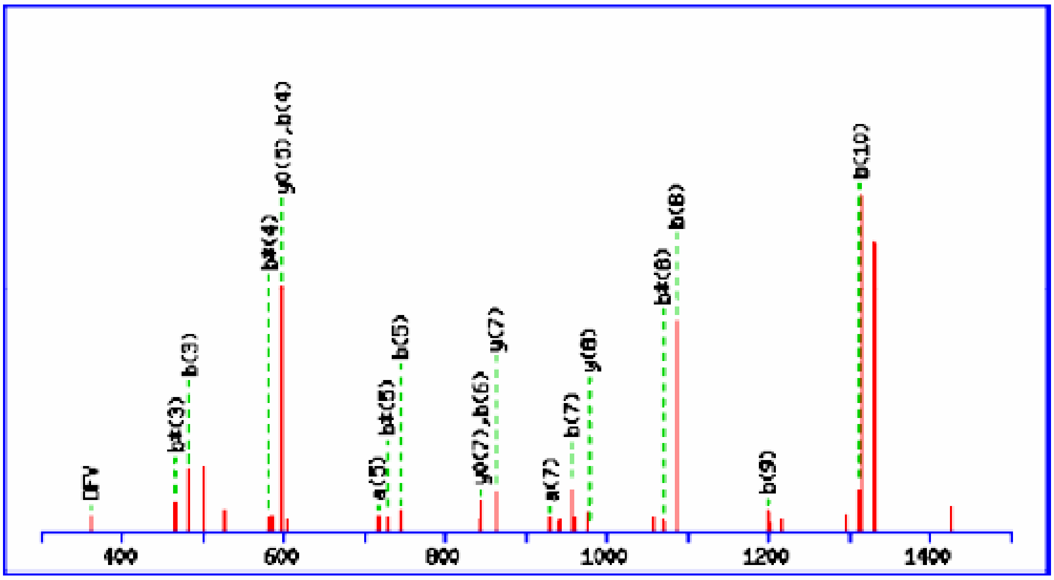

Monoisotopic mass of neutral peptide Mr(calc): 1458.7507
Fixed modifications: Carbamidomethyl (C) (apply to specified residues or termini only)
Ions Score: 59 **Expect:** 0.0073
Matches : 19/142 fragment ions using 26 most intense peaks (help)

#	Immon.	a	a*	a^0	b	b*	b^0	Seq.	y	y*	y^0	#
1	136.0757	136.0757			164.0706			Y				11
2	136.0757	299.1390			327.1339			Y	1296.6947	1279.6681	1278.6841	10
3	129.1135	455.2401	438.2136		*483.2350*	466.2085		R	1133.6313	1116.6048	1115.6208	9
4	88.0393	570.2671	553.2405	552.2565	*598.2620*	581.2354	580.2514	D	**977.5302**	960.5037	959.5197	8
5	120.0808	**717.3355**	700.3089	699.3249	*745.3304*	728.3039	727.3198	F	**862.5033**	845.4767	844.4927	7
6	72.0808	816.4039	799.3774	798.3933	*844.3988*	827.3723	826.3883	V	715.4349	698.4083	697.4243	6
7	86.0964	**929.4880**	912.4614	911.4774	*957.4829*	940.4563	939.4723	L	616.3665	599.3399	598.3559	5
8	102.0550	1058.5306	1041.5040	1040.5200	*1086.5255*	1069.4989	1068.5149	E	503.2824	486.2558	485.2718	4
9	86.0964	1171.6146	1154.5881	1153.6041	*1199.6095*	1182.5830	1181.5990	L	374.2398	357.2132		3
10	87.0553	1285.6575	1268.6310	1267.6470	*1313.6525*	1296.6259	1295.6419	N	261.1557	244.1292		2
11	101.1073							K	147.1128	130.0863		1

Seq	ya	yb	Seq	ya	yb	Seq	ya	yb
YR	292.1768	320.1717	YRD	407.2037	435.1987	YRDF	554.2722	582.2671
YRDFV	653.3406	681.3355	RD	244.1404	272.1353	RDF	391.2088	419.2037
RDFV	490.2772	518.2722	RDFVL	603.3613	631.3562	DF	235.1077	263.1026
DFV	334.1761	362.1710	DFVL	447.2602	475.2551	DFVLE	576.3028	604.2977
DFVLEL	689.3869	717.3818	FV	219.1492	247.1441	FVL	332.2333	360.2282
FVLE	461.2758	489.2708	FVLEL	574.3599	602.3548	FVLELN	688.4028	716.3978
VL	185.1648	213.1598	VLE	314.2074	342.2023	VLEL	427.2915	455.2864
VLELN	541.3344	569.3293	LE	215.1390	243.1339	LEL	328.2231	356.2180
LELN	442.2660	470.2609	EL	215.1390	243.1339	ELN	329.1819	357.1769
LN	200.1394	228.1343						

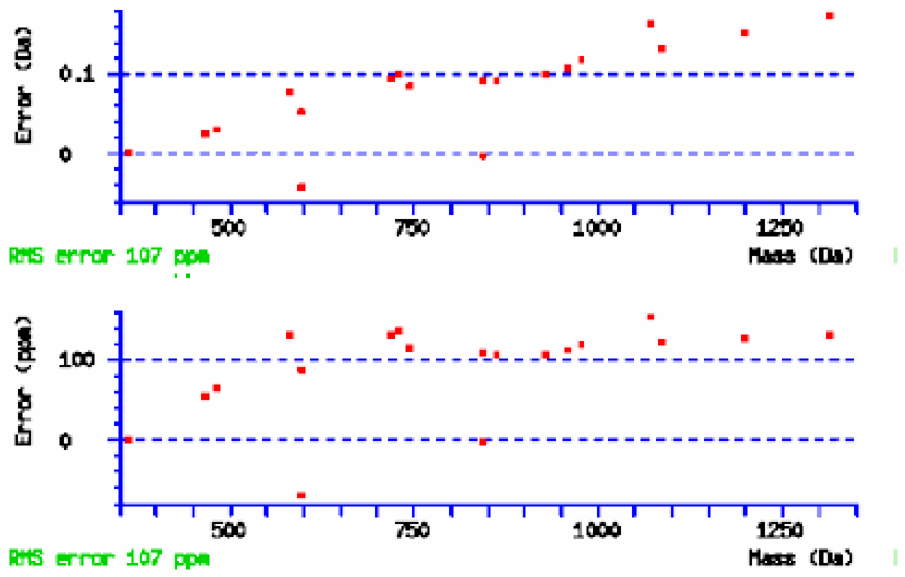

Spot 24. 6,7-Dimethyl-8-ribityllumazine synthase

Peptide View

MS/MS Fragmentation of SQKYEAILCIGAVIR
Found in gi|397174509, 6,7-dimethyl-8-ribityllumazine synthase, partial [Hyoscyamus albus]

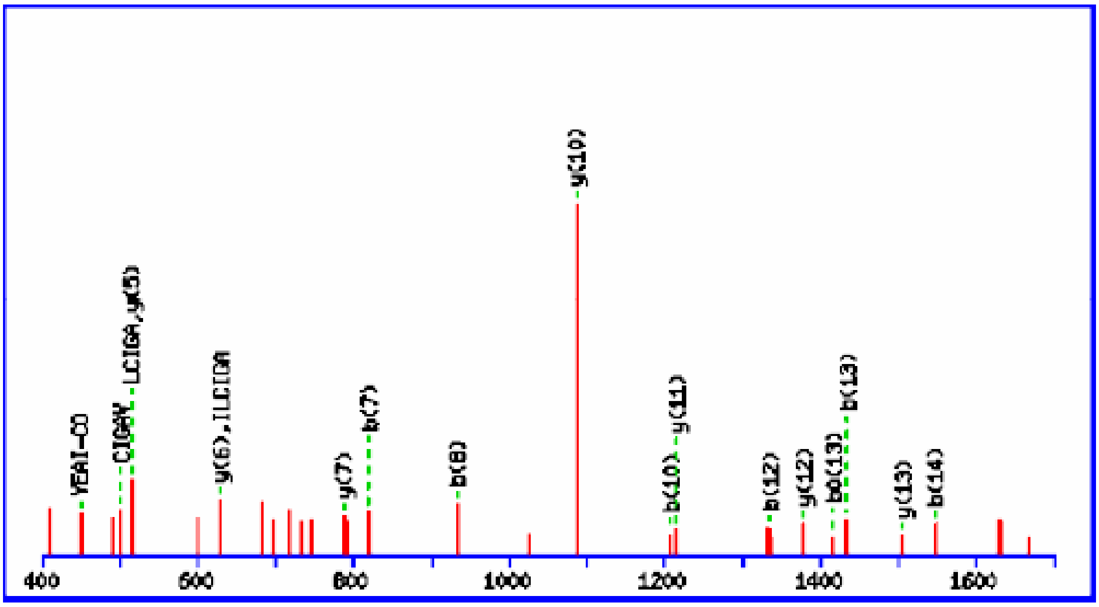

Monoisotopic mass of neutral peptide Mr(calc): 1719.9341
Fixed modifications: Carbamidomethyl (C) (apply to specified residues or termini only)
Ions Score: 55 **Expect:** 0.0088
Matches : 19/227 fragment ions using 27 most intense peaks (help)

#	Immon.	a	a*	a^0	b	b*	b^0	Seq.	y	y*	y^0	#
1	60.0444	60.0444		42.0338	88.0393		70.0287	S				15
2	101.0709	188.1030	171.0764	170.0924	216.0979	199.0713	198.0873	Q	1633.9094	1616.8829	1615.8989	14
3	101.1073	316.1979	299.1714	298.1874	344.1928	327.1663	326.1823	K	1505.8508	1488.8243	1487.8403	13
4	136.0757	479.2613	462.2347	461.2507	507.2562	490.2296	489.2456	Y	1377.7559	1360.7293	1359.7453	12
5	102.0550	608.3039	591.2773	590.2933	636.2988	619.2722	618.2882	E	1214.6926	1197.6660	1196.6820	11
6	44.0495	679.3410	662.3144	661.3304	707.3359	690.3093	689.3253	A	1085.6500	1068.6234		10
7	86.0964	792.4250	775.3985	774.4145	820.4199	803.3934	802.4094	I	1014.6128	997.5863		9
8	86.0964	905.5091	888.4825	887.4985	933.5040	916.4775	915.4934	L	901.5288	884.5022		8
9	133.0430	1065.5397	1048.5132	1047.5292	1093.5347	1076.5081	1075.5241	C	788.4447	771.4182		7
10	86.0964	1178.6238	1161.5973	1160.6132	1206.6187	1189.5922	1188.6082	I	628.4141	611.3875		6
11	30.0338	1235.6453	1218.6187	1217.6347	1263.6402	1246.6136	1245.6296	G	515.3300	498.3035		5
12	44.0495	1306.6824	1289.6558	1288.6718	1334.6773	1317.6508	1316.6667	A	458.3085	441.2820		4
13	72.0808	1405.7508	1388.7243	1387.7402	1433.7457	1416.7192	1415.7351	V	387.2714	370.2449		3
14	86.0964	1518.8349	1501.8083	1500.8243	1546.8298	1529.8032	1528.8192	I	288.2030	271.1765		2
15	129.1135							R	175.1190	158.0924		1

Seq	ya	yb	Seq	ya	yb	Seq	ya	yb
QK	229.1659	257.1608	QKY	392.2292	420.2241	QKYE	521.2718	549.2667
QKYEA	592.3089	620.3039	KY	264.1707	292.1656	KYE	393.2132	421.2082
KYEA	464.2504	492.2453	KYEAI	577.3344	605.3293	KYEAIL	690.4185	718.4134
YE	265.1183	293.1132	YEA	336.1554	364.1503	YEAI	449.2395	477.2344
YEAIL	562.3235	590.3184	EA	173.0921	201.0870	EAI	286.1761	314.1710
EAIL	399.2602	427.2551	EAILC	559.2908	587.2858	EAILCI	672.3749	700.3698
AI	157.1335	185.1285	AIL	270.2176	298.2125	AILC	430.2483	458.2432
AILCI	543.3323	571.3272	AILCIG	600.3538	628.3487	AILCIGA	671.3909	699.3858
IL	199.1805	227.1754	ILC	359.2111	387.2061	ILCI	472.2952	500.2901
ILCIG	529.3167	557.3116	ILCIGA	600.3538	628.3487	ILCIGAV	699.4222	727.4171
LC	246.1271	274.1220	LCI	359.2111	387.2061	LCIG	416.2326	444.2275
LCIGA	487.2697	515.2646	LCIGAV	586.3381	614.3330	LCIGAVI	699.4222	727.4171
CI	246.1271	274.1220	CIG	303.1485	331.1435	CIGA	374.1857	402.1806
CIGAV	473.2541	501.2490	CIGAVI	586.3381	614.3330	IG	143.1179	171.1128
IGA	214.1550	242.1499	IGAV	313.2234	341.2183	IGAVI	426.3075	454.3024
GA	101.0709	129.0659	GAV	200.1394	228.1343	GAVI	313.2234	341.2183
AV	143.1179	171.1128	AVI	256.2020	284.1969	VI	185.1648	213.1598

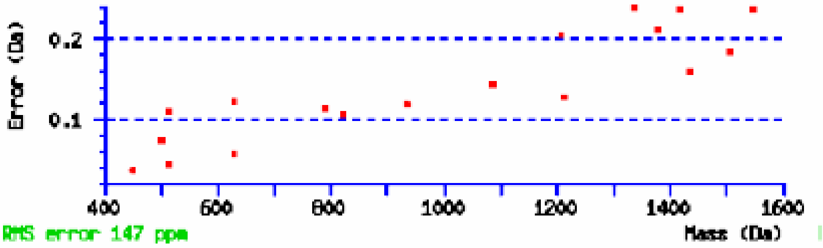

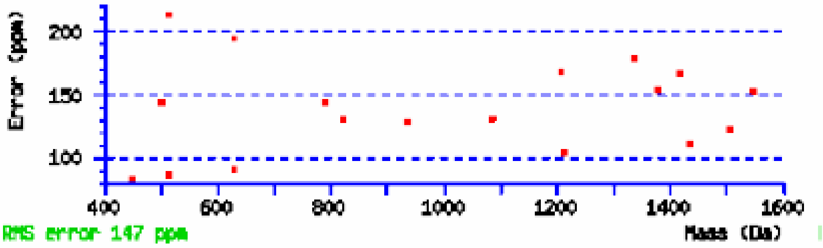

Spot 38. Predicted cationic peroxidase

Peptide View

MS/MS Fragmentation of **TFSKDFAESMIK**
Found in **gi|227581441**, FN041273 substractive cDNA library of Petunia hybrida, Mitchell diploid (W115)(EB-4) Petunia x hybrida cDNA clone drs21P0003P14_F.ab1 2007-08-10, mRNA sequence
Translated in frame 5 (nucleic acid sequence)

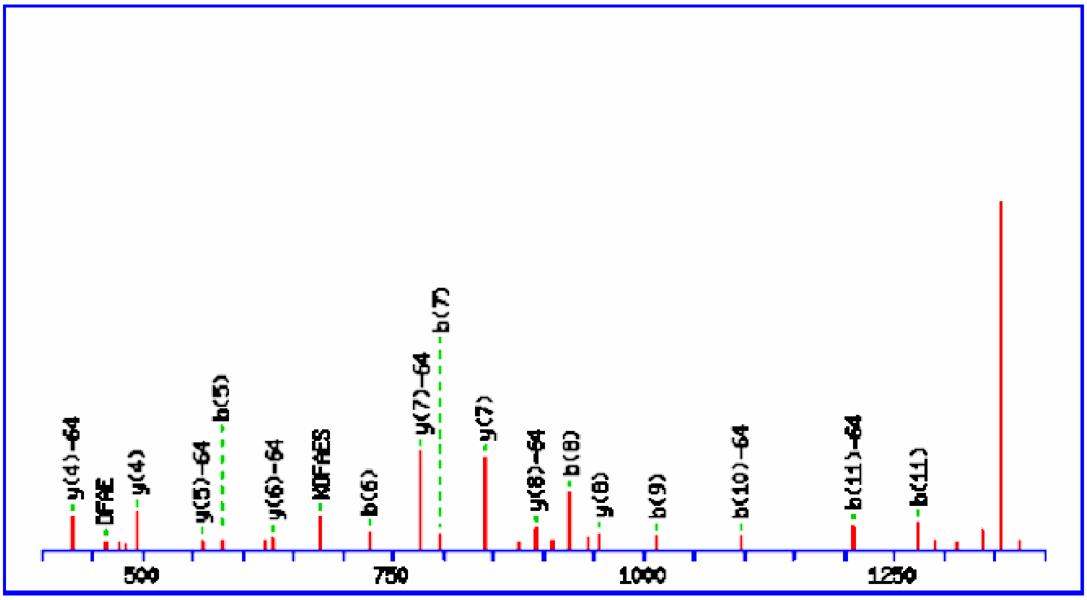

Monoisotopic mass of neutral peptide Mr(calc): 1418.6752
Fixed modifications: Carbamidomethyl (C) (apply to specified residues or termini only)
Variable modifications:
M10 : Oxidation (M), with neutral losses 63.9983(shown in table), 0.0000
Ions Score: 48 **Expect:** 0.055
Matches : 19/231 fragment ions using 25 most intense peaks (help)

#	Immon.	a	a*	a^0	b	b*	b^0	Seq.	y	y*	y^0	#
1	74.0600	74.0600		56.0495	102.0550		84.0444	T				12
2	120.0808	221.1285		203.1179	249.1234		231.1128	F	1254.6365	1237.6099	1236.6259	11
3	60.0444	308.1605		290.1499	336.1554		318.1448	S	1107.5681	1090.5415	1089.5575	10
4	101.1073	436.2554	419.2289	418.2449	464.2504	447.2238	446.2398	K	1020.5360	1003.5095	1002.5255	9
5	88.0393	551.2824	534.2558	533.2718	***579.2773***	562.2508	561.2667	D	**892.4411**	875.4145	874.4305	8
6	120.0808	698.3508	681.3243	680.3402	***726.3457***	709.3192	708.3352	F	**777.4141**	760.3876	759.4036	7
7	44.0495	769.3879	752.3614	751.3774	***797.3828***	780.3563	779.3723	A	**630.3457**	613.3192	612.3352	6
8	102.0550	898.4305	881.4040	880.4199	***926.4254***	909.3989	908.4149	E	**559.3086**	542.2821	541.2980	5
9	60.0444	985.4625	968.4360	967.4520	***1013.4575***	996.4309	995.4469	S	**430.2660**	413.2395	412.2554	4
10	56.0495	1068.4997	1051.4731	1050.4891	***1096.4946***	1079.4680	1078.4840	M	343.2340	326.2074		3
11	86.0964	1181.5837	1164.5572	1163.5732	***1209.5786***	1192.5521	1191.5681	I	260.1969	243.1703		2
12	101.1073							K	147.1128	130.0863		1

Seq	ya	yb	Seq	ya	yb	Seq	ya	yb
FS	207.1128	235.1077	FSK	335.2078	363.2027	FSKD	450.2347	478.2296
FSKDF	597.3031	625.2980	FSKDFA	668.3402	696.3352	SK	188.1394	216.1343
SKD	303.1663	331.1612	SKDF	450.2347	478.2296	SKDFA	521.2718	549.2667
SKDFAE	650.3144	678.3093	KD	216.1343	244.1292	KDF	363.2027	391.1976
KDFA	434.2398	462.2347	KDFAE	563.2824	591.2773	KDFAES	650.3144	678.3093
DF	235.1077	263.1026	DFA	306.1448	334.1397	DFAE	435.1874	463.1823
DFAES	522.2195	550.2144	DFAESM	605.2566	633.2515	FA	191.1179	219.1128
FAE	320.1605	348.1554	FAES	407.1925	435.1874	FAESM	490.2296	518.2245
FAESMI	603.3137	631.3086	AE	173.0921	201.0870	AES	260.1241	288.1190
AESM	343.1612	371.1561	AESMI	456.2453	484.2402	ES	189.0870	217.0819
ESM	272.1241	300.1190	ESMI	385.2082	413.2031	SM	143.0815	171.0764
SMI	256.1656	284.1605	MI	169.1335	197.1285			

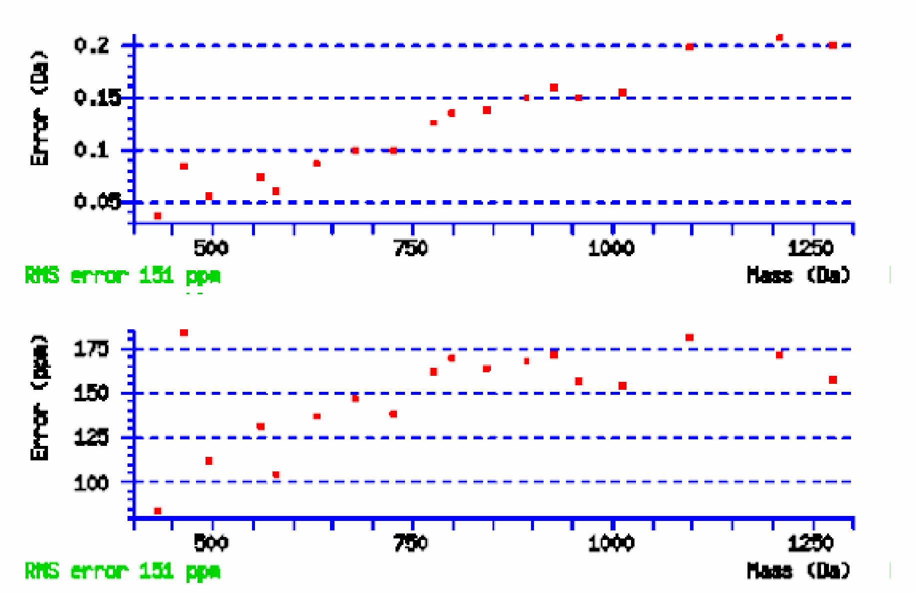

Spot 41. Fumarase

Peptide View

MS/MS Fragmentation of **SVEFKDIIK**
Found in **gi|190858454**, AGN_PNL220df1_b2.trimmed.seq AGN_PNL Nicotiana tabacum cDNA 5', mRNA sequence
Translated in frame 2 (nucleic acid sequence)

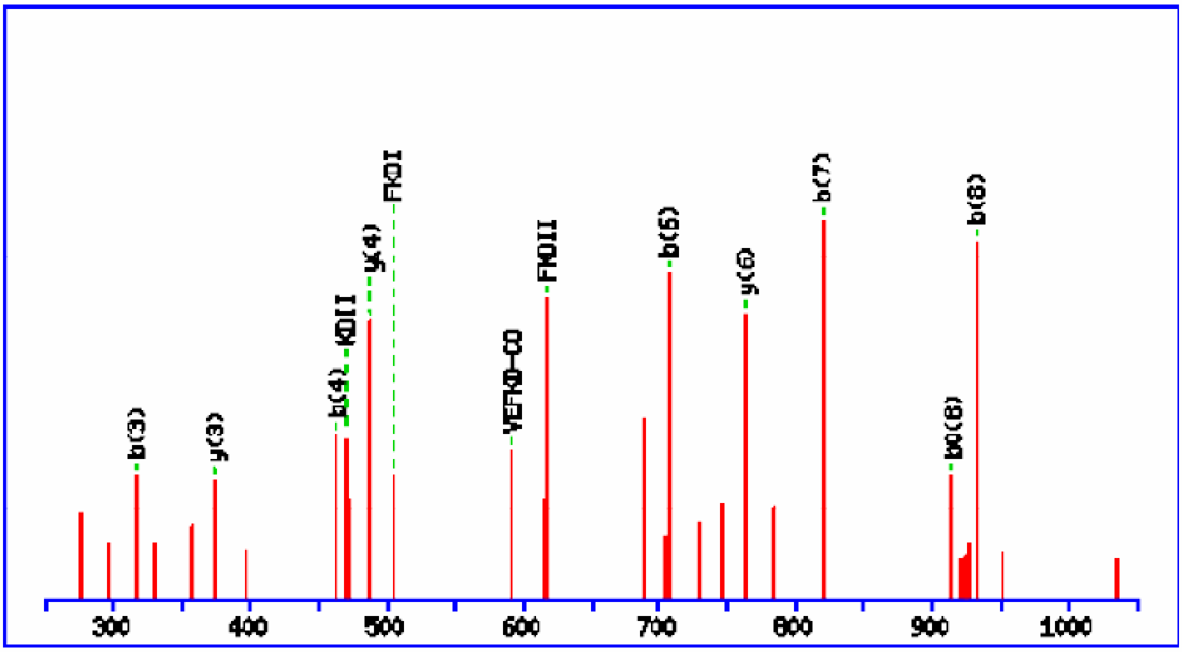

Monoisotopic mass of neutral peptide Mr(calc): 1077.6070
Fixed modifications: Carbamidomethyl (C) (apply to specified residues or termini only)
Ions Score: 56 **Expect:** 0.011
Matches : 16/106 fragment ions using 15 most intense peaks (help)

#	Immon.	a	a*	a^0	b	b*	b^0	Seq.	y	y*	y^0	#
1	60.0444	60.0444		42.0338	88.0393		70.0287	S				9
2	72.0808	159.1128		141.1022	187.1077		169.0972	V	991.5823	974.5557	973.5717	8
3	102.0550	288.1554		270.1448	*316.1503*		298.1397	E	892.5138	875.4873	874.5033	7
4	120.0808	435.2238		417.2132	*463.2187*		445.2082	F	**763.4713**	746.4447	745.4607	6
5	101.1073	563.3188	546.2922	545.3082	*591.3137*	574.2871	573.3031	K	616.4028	599.3763	598.3923	5
6	88.0393	678.3457	661.3192	660.3352	*706.3406*	689.3141	688.3301	D	**488.3079**	471.2813	470.2973	4
7	86.0964	791.4298	774.4032	773.4192	*819.4247*	802.3981	801.4141	I	**373.2809**	356.2544		3
8	86.0964	904.5138	887.4873	886.5033	*932.5088*	915.4822	914.4982	I	260.1969	243.1703		2
9	101.1073							K	147.1128	130.0863		1

Seq	ya	yb	Seq	ya	yb	Seq	ya	yb
VE	201.1234	229.1183	VEF	348.1918	376.1867	VEFK	476.2867	504.2817
VEFKD	591.3137	619.3086	EF	249.1234	277.1183	EFK	377.2183	405.2132
EFKD	492.2453	520.2402	EFKDI	605.3293	633.3243	FK	248.1757	276.1707
FKD	363.2027	391.1976	FKDI	476.2867	504.2817	FKDII	589.3708	617.3657
KD	216.1343	244.1292	KDI	329.2183	357.2132	KDII	442.3024	470.2973
DI	201.1234	229.1183	DII	314.2074	342.2023	II	199.1805	227.1754

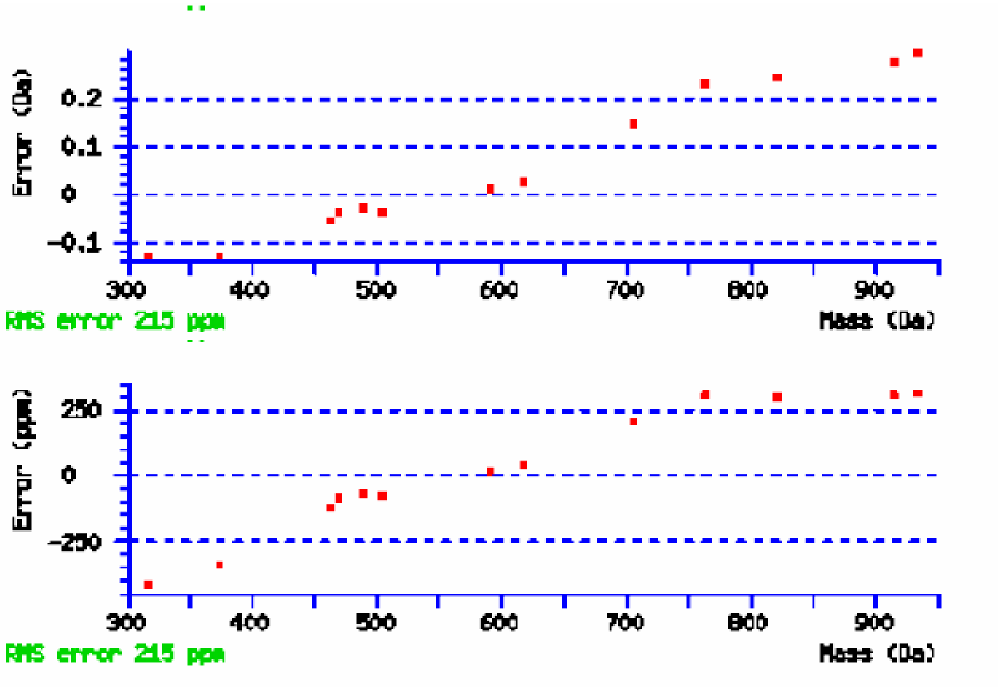

Spot 49. Soluble inorganic pyrophosphatase

Peptide View

MS/MS Fragmentation of ILYSSVVYPQNYGFIPR
Found in IPYR_SOLTU, Soluble inorganic pyrophosphatase OS=Solanum tuberosum GN=PPA PE=2 SV=1

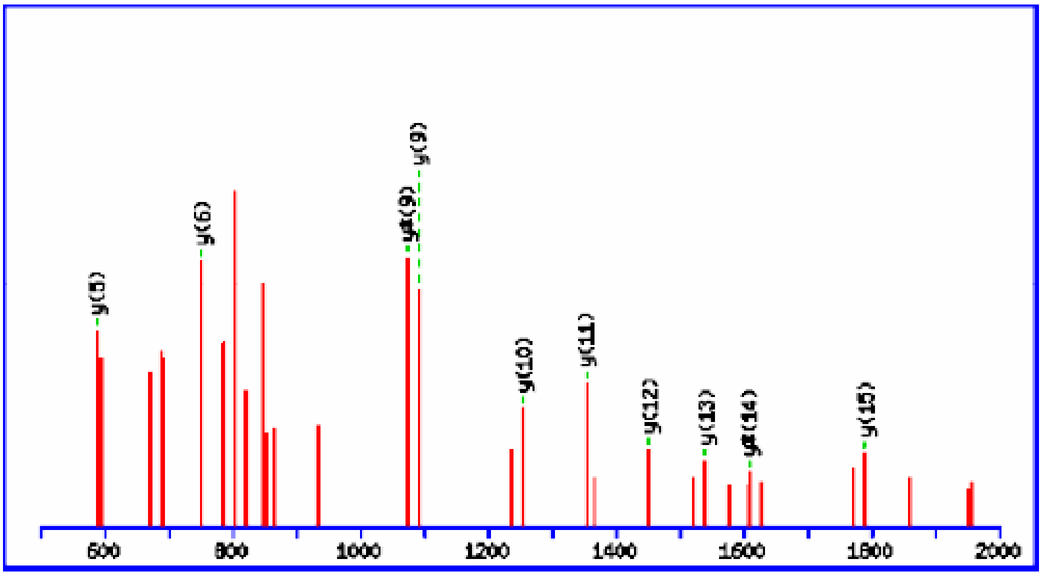

Monoisotopic mass of neutral peptide Mr(calc): 2015.0516
Fixed modifications: Carbamidomethyl (C) (apply to specified residues or termini only)
Ions Score: 58 **Expect:** 0.0014
Matches : 10/239 fragment ions using 14 most intense peaks (help)

#	Immon.	a	a*	a^0	b	b*	b^0	Seq.	y	y*	y^0	#
1	86.0964	86.0964			114.0913			I				17
2	86.0964	199.1805			227.1754			L	1902.9749	1885.9483	1884.9643	16
3	136.0757	362.2438			390.2387			Y	*1789.8908*	1772.8642	1771.8802	15
4	60.0444	449.2758		431.2653	477.2708		459.2602	S	1626.8275	1609.8009	1608.8169	14
5	60.0444	536.3079		518.2973	564.3028		546.2922	S	*1539.7954*	1522.7689	1521.7849	13
6	72.0808	635.3763		617.3657	663.3712		645.3606	V	*1452.7634*	1435.7369		12
7	72.0808	734.4447		716.4341	762.4396		744.4291	V	*1353.6950*	1336.6685		11
8	136.0757	897.5080		879.4975	925.5029		907.4924	Y	*1254.6266*	1237.6000		10
9	70.0651	994.5608		976.5502	1022.5557		1004.5451	P	*1091.5633*	1074.5367		9
10	101.0709	1122.6194	1105.5928	1104.6088	1150.6143	1133.5877	1132.6037	Q	994.5105	977.4839		8
11	87.0553	1236.6623	1219.6358	1218.6517	1264.6572	1247.6307	1246.6466	N	866.4519	849.4254		7
12	136.0757	1399.7256	1382.6991	1381.7151	1427.7205	1410.6940	1409.7100	Y	*752.4090*	735.3824		6
13	30.0338	1456.7471	1439.7205	1438.7365	1484.7420	1467.7155	1466.7314	G	*589.3457*	572.3191		5
14	120.0808	1603.8155	1586.7890	1585.8049	1631.8104	1614.7839	1613.7999	F	532.3242	515.2976		4
15	86.0964	1716.8996	1699.8730	1698.8890	1744.8945	1727.8679	1726.8839	I	385.2558	368.2292		3
16	70.0651	1813.9523	1796.9258	1795.9418	1841.9472	1824.9207	1823.9367	P	272.1717	255.1452		2
17	129.1135							R	175.1190	158.0924		1

Seq	ya	yb	Seq	ya	yb	Seq	ya	yb
LY	249.1598	277.1547	LYS	336.1918	364.1867	LYSS	423.2238	451.2187
LYSSV	522.2922	550.2871	LYSSVV	621.3606	649.3556	YS	223.1077	251.1026
YSS	310.1397	338.1347	YSSV	409.2082	437.2031	YSSVV	508.2766	536.2715
YSSVVY	671.3399	699.3348	SS	147.0764	175.0713	SSV	246.1448	274.1397
SSVV	345.2132	373.2082	SSVVY	508.2766	536.2715	SSVVYP	605.3293	633.3243
SV	159.1128	187.1077	SVV	258.1812	286.1761	SVVY	421.2445	449.2395
SVVYP	518.2973	546.2922	SVVYPQ	646.3559	674.3508	VV	171.1492	199.1441
VVY	334.2125	362.2074	VVYP	431.2653	459.2602	VVYPQ	559.3239	587.3188
VVYPQN	673.3668	701.3617	VY	235.1441	263.1390	VYP	332.1969	360.1918
VYPQ	460.2554	488.2504	VYPQN	574.2984	602.2933	YP	233.1285	261.1234
YPQ	361.1870	389.1819	YPQN	475.2300	503.2249	YPQNY	638.2933	666.2882
YPQNYG	695.3148	723.3097	PQ	198.1237	226.1186	PQN	312.1666	340.1615
PQNY	475.2300	503.2249	PQNYG	532.2514	560.2463	PQNYGF	679.3198	707.3148
QN	215.1139	243.1088	QNY	378.1772	406.1721	QNYG	435.1987	463.1936
QNYGF	582.2671	610.2620	QNYGFI	695.3511	723.3461	NY	250.1186	278.1135
NYG	307.1401	335.1350	NYGF	454.2085	482.2034	NYGFI	567.2926	595.2875
NYGFIP	664.3453	692.3402	YG	193.0972	221.0921	YGF	340.1656	368.1605
YGFI	453.2496	481.2445	YGFIP	550.3024	578.2973	GF	177.1022	205.0972
GFI	290.1863	318.1812	GFIP	387.2391	415.2340	FI	233.1648	261.1598
FIP	330.2176	358.2125	IP	183.1492	211.1441			

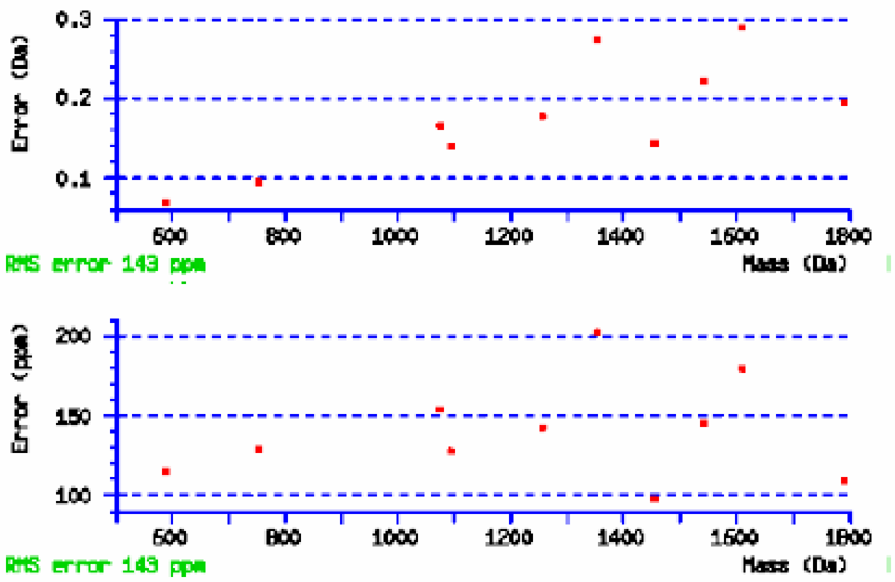

Spot 58.UDP-Glucose pyrophosphorylase

Peptide View

MS/MS Fragmentation of **SNPSNPSIELGPEFKK**
Found in **UGPA_SOLTU**, UTP--glucose-1-phosphate uridylyltransferase OS=Solanum tuberosum PE=1 SV=3

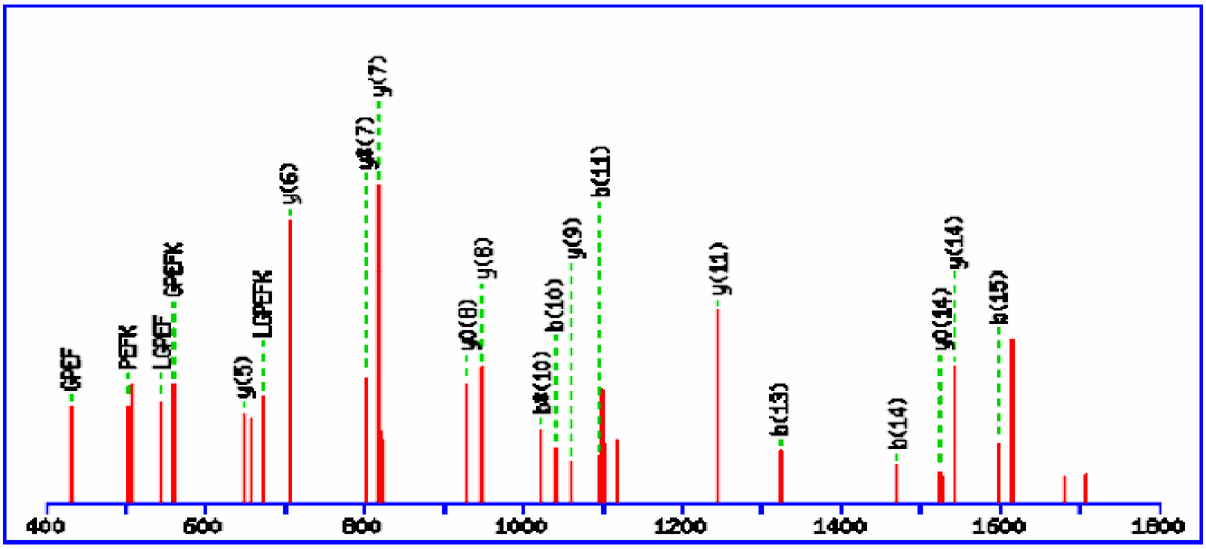


```
Monoisotopic mass of neutral peptide Mr(calc): 1742.8839
Fixed modifications: Carbamidomethyl (C) (apply to specified residues or termini only)
Ions Score: 51  Expect: 0.0073
Matches : 21/262 fragment ions using 32 most intense peaks   (help)
```

#	Immon.	a	a*	a^0	b	b*	b^0	Seq.	y	y*	y^0	#
1	60.0444	60.0444		42.0338	88.0393		70.0287	S				16
2	87.0553	174.0873	157.0608	156.0768	202.0822	185.0557	184.0717	N	1656.8592	1639.8326	1638.8486	15
3	70.0651	271.1401	254.1135	253.1295	299.1350	282.1084	281.1244	P	*1542.8162*	1525.7897	1524.8057	14
4	60.0444	358.1721	341.1456	340.1615	386.1670	369.1405	368.1565	S	1445.7635	1428.7369	1427.7529	13
5	87.0553	472.2150	455.1885	454.2045	500.2099	483.1834	482.1994	N	1358.7314	1341.7049	1340.7209	12
6	70.0651	569.2678	552.2412	551.2572	597.2627	580.2362	579.2521	P	*1244.6885*	1227.6620	1226.6780	11
7	60.0444	656.2998	639.2733	638.2893	684.2947	667.2682	666.2842	S	1147.6358	1130.6092	1129.6252	10
8	86.0964	769.3839	752.3573	751.3733	797.3788	780.3523	779.3682	I	*1060.6037*	1043.5772	1042.5932	9
9	102.0550	898.4265	881.3999	880.4159	926.4214	909.3948	908.4108	E	*947.5197*	930.4931	929.5091	8
10	86.0964	1011.5105	994.4840	993.5000	*1039.5055*	1022.4789	1021.4949	L	*818.4771*	801.4505	800.4665	7
11	30.0338	1068.5320	1051.5055	1050.5214	*1096.5269*	1079.5004	1078.5164	G	*705.3930*	688.3665	687.3824	6
12	70.0651	1165.5848	1148.5582	1147.5742	1193.5797	1176.5531	1175.5691	P	*648.3715*	631.3450	630.3610	5
13	102.0550	1294.6274	1277.6008	1276.6168	*1322.6223*	1305.5957	1304.6117	E	551.3188	534.2922	533.3082	4
14	120.0808	1441.6958	1424.6692	1423.6852	*1469.6907*	1452.6641	1451.6801	F	422.2762	405.2496		3
15	101.1073	1569.7907	1552.7642	1551.7802	*1597.7857*	1580.7591	1579.7751	K	275.2078	258.1812		2
16	101.1073							K	147.1128	130.0863		1

Seq	ya	yb	Seq	ya	yb	Seq	ya	yb
NP	184.1081	212.1030	NPS	271.1401	299.1350	NPSN	385.1830	413.1779
NPSNP	482.2358	510.2307	NPSNPS	569.2678	597.2627	NPSNPSI	682.3519	710.3468
PS	157.0972	185.0921	PSN	271.1401	299.1350	PSNP	368.1928	396.1878
PSNPS	455.2249	483.2198	PSNPSI	568.3089	596.3039	PSNPSIE	697.3515	725.3464
SN	174.0873	202.0822	SNP	271.1401	299.1350	SNPS	358.1721	386.1670
SNPSI	471.2562	499.2511	SNPSIE	600.2988	628.2937	NP	184.1081	212.1030
NPS	271.1401	299.1350	NPSI	384.2241	412.2191	NPSIE	513.2667	541.2617
NPSIEL	626.3508	654.3457	NPSIELG	683.3723	711.3672	PS	157.0972	185.0921
PSI	270.1812	298.1761	PSIE	399.2238	427.2187	PSIEL	512.3079	540.3028
PSIELG	569.3293	597.3243	PSIELGP	666.3821	694.3770	SI	173.1285	201.1234
SIE	302.1710	330.1660	SIEL	415.2551	443.2500	SIELG	472.2766	500.2715
SIELGP	569.3293	597.3243	SIELGPE	698.3719	726.3668	IE	215.1390	243.1339
IEL	328.2231	356.2180	IELG	385.2445	413.2395	IELGP	482.2973	510.2922
IELGPE	611.3399	639.3348	EL	215.1390	243.1339	ELG	272.1605	300.1554
ELGP	369.2132	397.2082	ELGPE	498.2558	526.2508	ELGPEF	645.3243	673.3192
LG	143.1179	171.1128	LGP	240.1707	268.1656	LGPE	369.2132	397.2082
LGPEF	516.2817	544.2766	LGPEFK	644.3766	672.3715	GP	127.0866	155.0815
GPE	256.1292	284.1241	GPEF	403.1976	431.1925	GPEFK	531.2926	559.2875
PE	199.1077	227.1026	PEF	346.1761	374.1710	PEFK	474.2711	502.2660
EF	249.1234	277.1183	EFK	377.2183	405.2132	FK	248.1757	276.1707

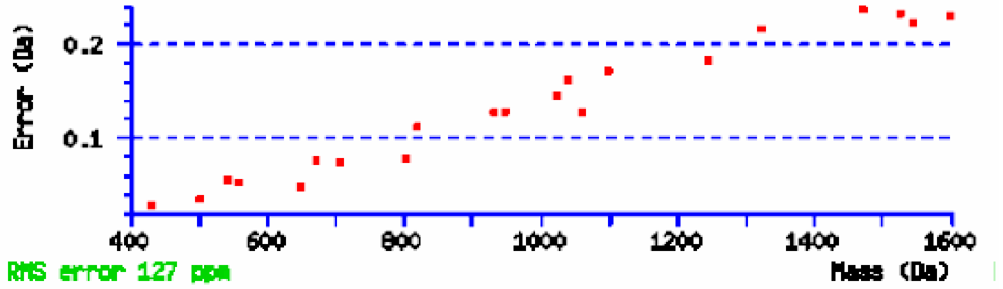

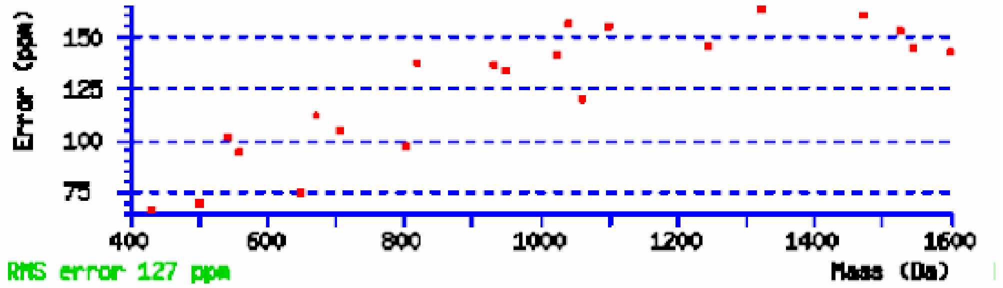

Supplement: Supplementary file 2 [file DataSheet2.PDF]
